# Inferring and evaluating satellite-based constraints on NO_*x*_ emissions estimates in air quality simulations

**DOI:** 10.5194/acp-22-15981-2022

**Published:** 2022-12-20

**Authors:** James D. East, Barron H. Henderson, Sergey L. Napelenok, Shannon N. Koplitz, Golam Sarwar, Robert Gilliam, Allen Lenzen, Daniel Q. Tong, R. Bradley Pierce, Fernando Garcia-Menendez

**Affiliations:** 1 Department of Civil, Construction, and Environmental Engineering, North Carolina State University, Raleigh, NC 27606, USA; 2 Oak Ridge Institute for Science and Education, Office of Research and Development, U.S. Environmental, Protection Agency, Research Triangle Park, NC 27711, USA; 3 U.S. Environmental Protection Agency, Research Triangle Park, NC 27711, USA; 4 Space Science and Engineering Center, University of Wisconsin-Madison, Madison, WI 53706, USA; 5 Department of Atmospheric, Oceanic and Earth Sciences, George Mason University, Fairfax, VA 22030, USA

## Abstract

Satellite observations of tropospheric NO_2_ columns can provide top-down observational constraints on emissions estimates of nitrogen oxides (NO_*x*_). Mass-balance-based methods are often applied for this purpose but do not isolate near-surface emissions from those aloft, such as lightning emissions. Here, we introduce an inverse modeling framework that couples satellite chemical data assimilation to a chemical transport model. In the framework, satellite-constrained emissions totals are inferred using model simulations with and without data assimilation in the iterative finite-difference mass-balance method. The approach improves the finite-difference mass-balance inversion by isolating the near-surface emissions increment. We apply the framework to separately estimate lightning and anthropogenic NO_*x*_ emissions over the Northern Hemisphere for 2019. Using overlapping observations from the Ozone Monitoring Instrument (OMI) and the Tropospheric Monitoring Instrument (TROPOMI), we compare separate NO_*x*_ emissions inferences from these satellite instruments, as well as the impacts of emissions changes on modeled NO_2_ and O_3_. OMI inferences of anthropogenic emissions consistently lead to larger emissions than TROPOMI inferences, attributed to a low bias in TROPOMI NO_2_ retrievals. Updated lightning NO_*x*_ emissions from either satellite improve the chemical transport model’s low tropospheric O_3_ bias. The combined lighting and anthropogenic emissions updates improve the model’s ability to reproduce measured ozone by adjusting natural, long-range, and local pollution contributions. Thus, the framework informs and supports the design of domestic and international control strategies.

## 1 Introduction

Tropospheric nitrogen oxides (NO_*x*_), nitric oxide (NO) and nitrogen dioxide (NO_2_), harm human health (Anenberg et al., 2018; Murray et al., 2020) and play a key role in the formation of important secondary atmospheric pollutants, such as O_3_ (Jacob, 2000). NO_*x*_ is emitted to the troposphere primarily by anthropogenic combustion processes, but natural sources, including soil, lightning, and wildfires, also contribute to the atmospheric NO_*x*_ budget (Jacob, 1999). Accurate NO_*x*_ emissions are a critical component of local- to global-scale atmospheric chemistry simulations. On hemispheric scales, realistically representing the formation and intercontinental transport of O_3_ with models requires adequate global emissions inventories (Itahashi et al., 2020; Zhang et al., 2016, 2008; Verstraeten et al., 2015; Mathur et al., 2017). In regional air quality simulations, which commonly rely on hemispheric or global models for chemical boundary conditions, the relative contribution of long-range pollutant transport to ground-level O_3_ concentrations has grown in many areas as O_3_ precursor emissions have decreased in the US and other high-income countries (HICs) (McDuffie et al., 2020; Jaffe et al., 2018; Simon et al., 2015). As a result, air quality management policies, often informed by regional modeling, are strengthened by accurate and up-to-date global NO_*x*_ emissions inventories. However, compilation of bottom-up regional and global emissions inventories, developed from source- and location-specific emissions factors and activity data, is time- and labor-intensive and can be hindered by limited data. As a result, bottom-up inventories lag behind emissions changes and are often incomplete. Uncertainties in bottom-up emissions estimates are particularly large for lower–middle-income countries (McDuffie et al., 2020; Elguindi et al., 2020) and remain significant for HICs (Day et al., 2019).

Satellite observations of NO_2_ can bridge temporal gaps in emissions estimates (Tong et al., 2016, 2015) and constrain uncertainty in emissions inventories through inverse modeling (e.g., Lamsal et al., 2011; Goldberg et al., 2021; de Foy and Schauer, 2022). Several methods have been applied to develop top-down emissions estimates using satellite observations and atmospheric models, each carrying advantages and limitations (Elguindi et al., 2020). Adjoint-based methods can provide precise emissions updates but require significant computational resources (e.g., Qu et al., 2017, 2019; Muller and Stavrakou, 2005; Kurokawa et al., 2009; Cooper et al., 2017; Zhang et al., 2019; Y. Wang et al., 2020). Similarly, Kalman filtering and related approaches have been used but are computationally intensive (e.g., Napelenok et al., 2008; Ding et al., 2020, 2015; Mijling and van der A, 2012; Miyazaki and Eskes, 2013; Miyazaki et al., 2017, 2012a, b; Sekiya et al., 2021). Mass-balance inversion approaches, which scale model emissions by directly comparing model estimates and satellite observations, were introduced by Martin et al. (2003), updated by Lamsal et al. (2011), and have been widely used in research and forecasting (e.g., Boersma et al., 2015; Itahashi et al., 2019; Li et al., 2018; Visser et al., 2019; Zhu et al., 2021; Cooper et al., 2017). Although lower computational costs allow the finite-difference mass-balance (FDMB) approach (Lamsal et al., 2011) to readily update emissions, the method is subject to an emissions-smearing effect (e.g., Cooper et al., 2017), which can cause emissions updates to be spatially misallocated. Since FDMB uses satellite observations directly, near-surface NO_2_ bias cannot be isolated from biases in the middle and upper troposphere, which obscures the surface emissions inference. Further, applications often rely on a single inversion from a single satellite, although available satellite products have been shown to have significant biases. For example, early versions of the Tropospheric Monitoring Instrument (TROPOMI) NO_2_ product showed a low bias in urban areas when compared against ground-based and airborne spectrometer measurements (Judd et al., 2020; Verhoelst et al., 2021), and the Ozone Monitoring Instrument (OMI) NO_2_ product has been reported to differ with measurements by ±20% (Lamsal et al., 2014). The impact of biases in satellite-based NO_2_ data on mass-balance inversions has not been fully explored despite the wide use of the method to scale NO_*x*_ emissions. Minimizing biases in anthropogenic emissions inferences and understanding the potential for them to propagate to emissions updates are needed to improve mass-balance-based inversions.

Here, we introduce a modeling framework that couples satellite chemical data assimilation to the Community Multiscale Air Quality model (CMAQ) and applies an iterative FDMB inversion to estimate NO_*x*_ emissions in the Northern Hemisphere. The framework provides observational constraints to improve emissions estimates in areas where emissions are highly uncertain, at a lower computational cost relative to adjoint- and Kalman-filter-based approaches. We apply the framework in an iterative assimilation to infer 2019 NO_*x*_ emissions, the first complete year in which OMI and TROPOMI records overlap. In contrast to traditional FDMB approaches, which directly compares modeled and observed columns, our framework improves the FDMB method by first assimilating satellite-retrieved NO_2_ and then performing the inversion by comparing model simulations with and without assimilation. In the assimilation step, updates to model concentrations are vertically allocated to model layers. As a result, assimilating the observed column allows the inversion framework to use only the near-surface portion of the model column in the FDMB inversion, minimizing influences from the upper troposphere and extending the framework proposed by Lamsal et al. (2011). In addition, our analysis compares independent inversions which separately use OMI or TROPOMI NO_2_ data. We show that the inverse emissions produced by this framework influence the representation of intercontinental O_3_ transport to the US, offering an opportunity to improve chemical boundary conditions in policy-relevant regional-scale air quality simulations.

## 2 Methods

**Figure 1.**
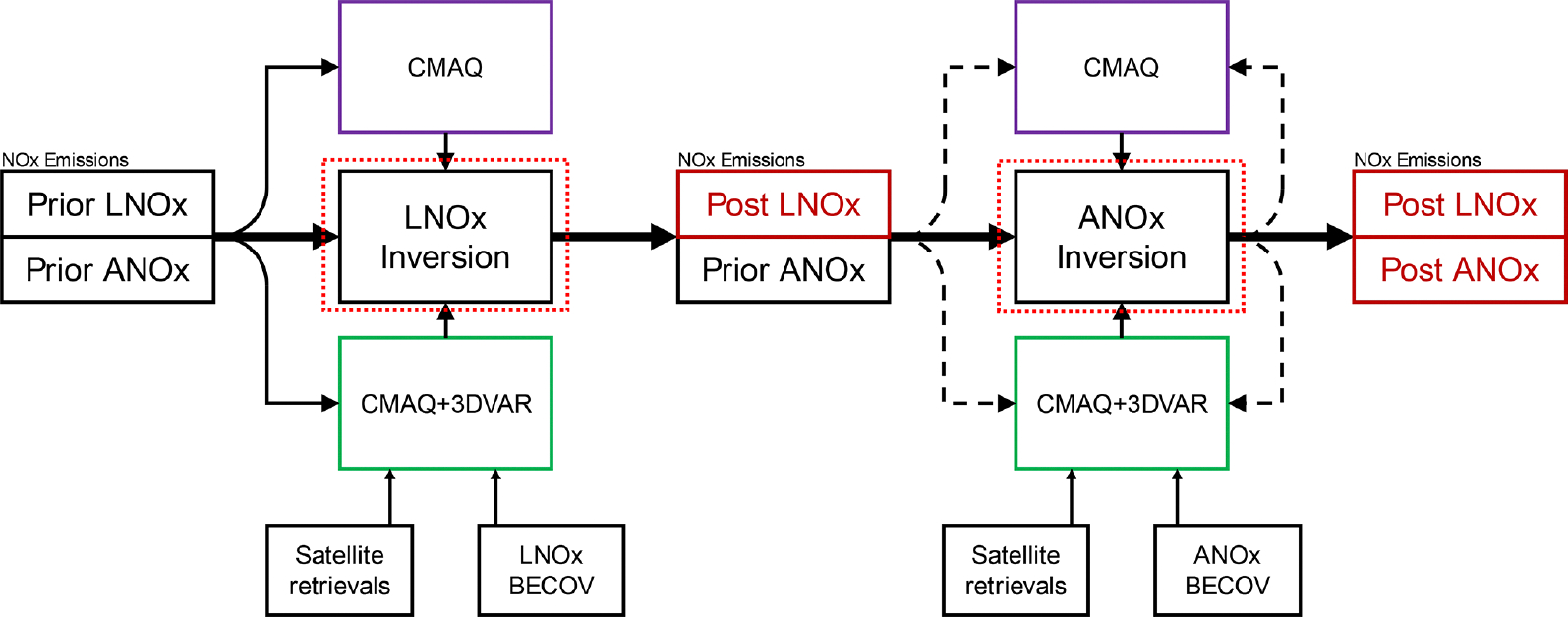
NO_*x*_ emissions inversion framework. Lightning NO_*x*_ (LNO_*x*_) emissions are updated in the first step. Then, anthropogenic NO_*x*_ (ANO_*x*_) emissions are updated iteratively. CMAQ boxes represent air quality simulations without chemical data assimilation, and CMAQ+3DVAR boxes represent air quality model simulations with chemical data assimilation. Satellite NO_2_ retrievals and a background error covariance (BECOV) are inputs to the chemical data assimilation, described in Sect. 2.3. Red dotted lines around the inversion boxes correspond to the red dotted lines in Fig. 3, which details the inversion algorithm. Dashed black emissions input lines around the ANO_*x*_ inversion represent the iterative process. Iteration and convergence criteria are described in Sect. 2.6.

We develop a framework to update NO_*x*_ emissions estimates using the CMAQ chemical transport air quality model (Byun and Schere, 2006), 3D variational (3DVAR) chemical data assimilation (Sandu and Chai, 2011), and space-based NO_2_ observations. We apply the framework to estimate 2019 lightning and anthropogenic NO_*x*_ emissions and compare ground- and space-based NO_2_ observations to model simulations using the prior emissions (inventory before the framework is applied) and posterior emissions (inventory after the framework is applied) to assess the impact of the updates. Figure 1 provides an overview of the framework, in which lightning NO_*x*_ (LNO_*x*_) emissions and anthropogenic NO_*x*_ (ANO_*x*_) emissions are updated separately.

### 2.1 Satellite data

We use NO_2_ tropospheric column observations from the National Aeronautics and Space Administration’s (NASA’s) OMI and from the Royal Netherlands Meteorological Institute’s (KNMI’s) TROPOMI instruments in the inversion framework. TROPOMI was launched in October 2017 and provides 7.2 × 3.6 km^2^ resolution NO_2_ retrievals, upgraded to 5.6×3.6 km^2^ resolution in August 2019 (Van Geffen et al., 2020; Veefkind et al., 2012). TROPOMI’s sunsynchronous polar orbit crosses the Equator at approximately 13:30 local time (LT), allowing the instrument to achieve global coverage in one day. We assimilate the Level-2 tropospheric slant column retrieved from NASA’s Earth Science Data Systems program (https://www.earthdata.nasa.gov/, last access: 9 December 2022). The data product is described in the Algorithm and Theoretical Basis Document (ATBD) for TROPOMI NO_2_ (Van Geffen et al., 2019). We only consider TROPOMI observations with a quality flag greater than 0.5 and a cloud fraction lower than 30 % in the assimilation, following data product recommendations (Eskes et al., 2019). We use the latest publicly available versions of the TROPOMI retrieval for 2019 (versions 1.2.2 to 1.3.2) at the time of the analysis. Version 1.3 introduced updates to cloud processing that decreased noisy hotspots and broadened the range of acceptable air mass factors (Eskes et al., 2021). Information about the updates applied in each version and the dates on which updates were applied is given in Eskes et al. (2021). A research version with an updated retrieval applied to 2019 observations has been developed (Van Geffen et al., 2022) but was not yet standard and was not available at the time of this analysis. We discuss the impact of these latest updates in Sect. 3.3.

OMI, on board the Aura satellite launched in 2004, provides tropospheric NO_2_ vertical and slant column retrievals with a resolution of 13 × 24 km^2^ near nadir in a sunsynchronous polar orbit, with a local Equator crossing time of 13:45LT. Global coverage is achieved in 2 d. We use the NASA Goddard Space Flight Center (GSFC) Level-2 NO_2_ product (Krotkov et al., 2019b). OMI was impacted by a row anomaly beginning in 2008, reducing the number of usable pixels in the OMI retrieval (Boersma et al., 2018). We include only pixels with a cloud fraction lower than 30 % and a summary quality flag of 0. Detailed information about the NO_2_ data product is included in the OMI ATBD (Chance, 2002) and in Krotkov et al. (2019a).

**Figure 2.**
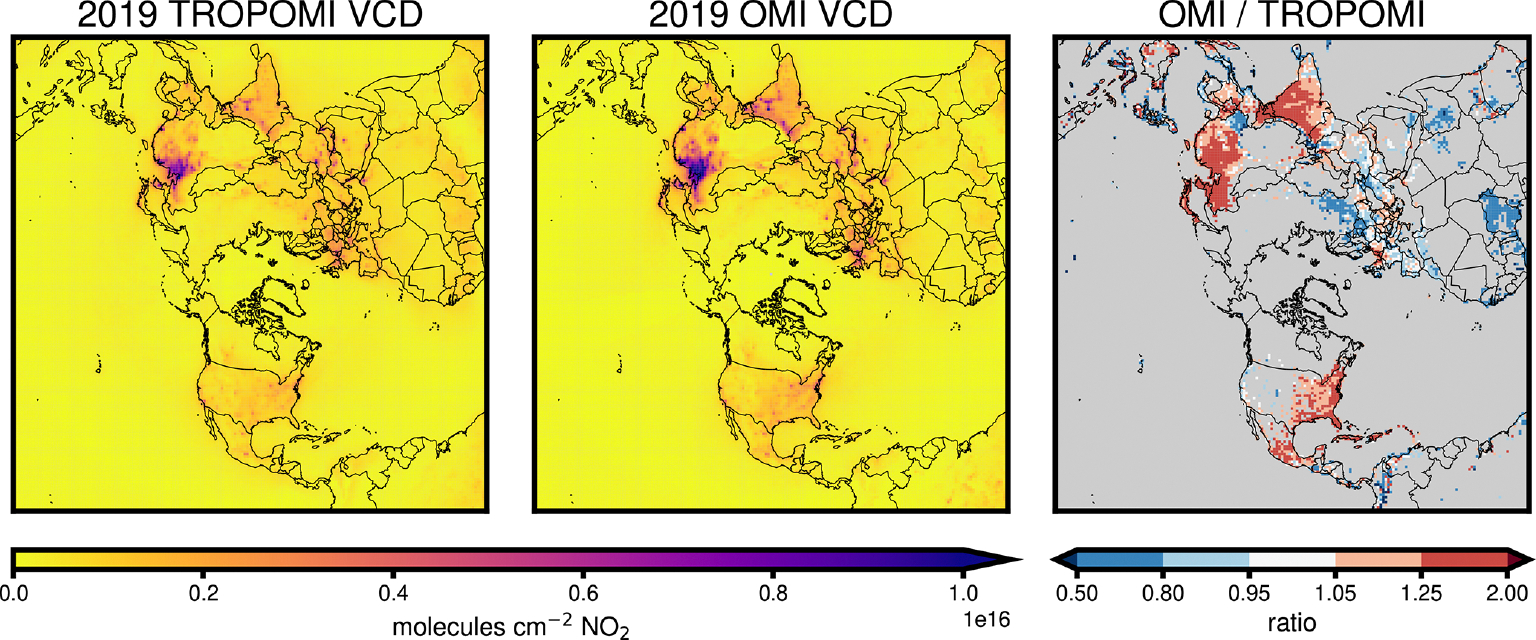
2019 annual average TROPOMI and OMI vertical NO_2_ vertical column densities, with CMAQ NO_2_ profiles applied, and the ratio between them. Column density ratios are only shown for the grid cells where NO_*x*_ emissions updates are applied in the emissions inversion.

A low bias has been noted in the versions of TROPOMI NO_2_ used for this study (Judd et al., 2020; Verhoelst et al., 2021). Although TROPOMI NO_2_ retrievals from 2019 have been reprocessed with retrieval version 2.3.1, resulting in an improvement of the bias (Eskes et al., 2021), these reprocessed datasets were not yet available at the time this analysis was conducted. Figure 2 compares TROPOMI and OMI tropospheric vertical column densities (VCDs) for 2019, regridded to the CMAQ grid used. For the VCDs shown in the figure, we remove the effect of the assumed vertical profile of NO_2_ from the original satellite product by recalculating the VCDs with the NO_2_ vertical profile simulated by CMAQ. In the results, we discuss the low bias in TROPOMI data and explore its impact on emissions inversions.

### 2.2 Hemispheric air quality modeling

Model simulations in the inversion framework were completed for January–December 2019 using CMAQ v5.3.2 (Appel et al., 2021; U.S. EPA Office of Research and Development, 2020). CMAQ has been used to simulate air quality over the Northern Hemisphere and has been shown to adequately capture chemical composition against observations (Mathur et al., 2017). Model inputs and satellite observations are summarized in Table 1. Simulations, designed to capture continental-scale pollutant transport, cover the Northern Hemisphere with 108 km horizontal grid spacing and a 44-layer vertical structure reaching 50 hPa (Mathur et al., 2017). The simulations use version CB6r3 of the Carbon Bond 6 chemical mechanism (Luecken et al., 2019), the AERO7 aerosol module (Xu et al., 2018) and updated halogen chemistry (Kang et al., 2021). Anthropogenic emissions are modeled using representative day-of-week emissions that change month to month. Representative-day emissions are created by averaging data from the prior emissions inventory on a day-of-week basis by month. For each day of the week and each month, there is a unique hourly emissions file that is used for every matching day of the week in that month. As a result, diurnal and weekly patterns are captured in the emissions, while daily variations that are specific to the prior emissions inventory year are averaged. The prior emissions inventory relies on the best available emissions data at the time of the study. Anthropogenic emissions for North America are from the U.S. Environmental Protection Agency’s (EPA) 2017 National Emissions Inventory (NEI) modeling platform (Adams, 2020). Emissions in China are for the year 2015 (Zhao et al., 2018), and emissions for the rest of the hemisphere are based on the Hemispheric Transport of Air Pollution (HTAP) version 2, projected from their original 2010 date to 2014, with scaling factors from the Community Emissions Data System (CEDS). To initialize the 2019 prior and posterior simulations and to reduce the impact of chemical initial conditions on the results, we use a 1-year spin-up period not considered for the analyses. CMAQ model runs are driven by meteorology from a retrospective hemispheric simulation using the Weather Research and Forecasting (WRF) model (Skamarock et al., 2008) version 4.1.1, configured following Mathur et al. (2017) and Xing et al. (2015).

### 2.3 Chemical data assimilation in CMAQ

We adjust modeled NO_2_ concentrations using satellite observations by coupling the CMAQ model to a data assimilation model, the National Centers for Environmental Prediction (NCEP) Gridpoint Statistical Interpolation (GSI) program version 3.3 (Shao et al., 2016). GSI performs 3D variational (3DVAR) data assimilation by minimizing the cost function, J:

(1) $$J=\frac{1}{2}\left[x^{T}B^{-1}x+(H(x)-y{)}^{T}R^{-1}(H(x)-y)\right],$$

where y is the observation innovation $y=y_{o}-H\left(x_{b}\right),\;x$ is the analysis increment $x=x_{a}-x_{b},$ $x_{a}$ is the analysis field (NO_2_ concentration after application of chemical data assimilation), $x_{b}$ is the model background (the simulated NO_2_ concentration before application of chemical data assimilation), $y_{o}$ is the satellite observations, B is the background error covariance matrix, R is the observation error matrix, and H is the observation operator. To compute the difference between the model column $\left(x_{b}\right)$ and the satellite column $\left(y_{o}\right)$, the observation operator H is applied, which transforms the model background to the form of the satellite observations. For TROPOMI data, the averaging kernel is first converted to scattering weights as

(2) $$w(z)=A(z)\times M_{\text{total}},$$

where A(z) is the vertically resolved TROPOMI averaging kernel for level $z,\;M_{\text{total}}$ is the air mass factor provided with the satellite data, and w(z) is the vertically resolved scattering weights. Scattering weights accompany the OMI NO_2_ data product, so this step is not needed to assimilate OMI data. Scattering weights are then applied to compute the model slant column as

(3) $$\Omega_{s}^{m}=\sum_{z}\;\Omega_{v}^{m}\left(z\right)w\left(z\right),\;z\leq z_{\text{tropopause}},$$

where $\Omega_{v}^{m}(z)$ is the model partial vertical column in the troposphere, interpolated to the satellite grid, and $\Omega_{s}^{m}$ is the model tropospheric slant column density (SCD). The difference between the modeled and observed slant columns, or the observation innovation y in Eq. (1), is estimated as

(4) $${\Omega^{\prime }}_{s}=\Omega_{v}^{o}M_{\text{trop}}-\Omega_{s}^{m},$$

where ${\Omega^{\prime }}_{s}$ is the analysis increment, $\Omega_{v}^{o}$ is the satellite tropospheric VCD, and $M_{\text{trop}}$ is the tropospheric air mass factor, distributed with the satellite data. We eliminate the influence of the a priori satellite vertical profile by computing the analysis increment with the modeled and observed SCD, which, unlike the VCD, does not rely on the a priori vertical NO_2_ profile assumed by the satellite.

We compute B using the Generalized Background Error covariance matrix model (GENBE v2.0) (Descombes et al., 2015), which models background errors by comparing a free-running simulation and a simulation with either lightning or anthropogenic NO_*x*_ emissions perturbed. We use GENBE with the prior simulation and a simulation with a uniform −15 % perturbation to LNO_*x*_ to create three-dimensional background errors in the upper troposphere for the LNO_*x*_ assimilation. After updating LNO_*x*_ emissions (as described in Sect. 2.5), we create three-dimensional background errors in the boundary layer for the anthropogenic NO_*x*_ assimilation by using GENBE with the LNO_*x*_ posterior simulation and a simulation with a −15 % perturbation to surface anthropogenic NO_*x*_ emissions. Observation error R is provided with the satellite data.

Online coupling between GSI and CMAQ was developed in this study to perform the assimilation. At each model time step in which a satellite observation is available, the CMAQ model simulation is paused, and 3DVAR assimilation is performed. The CMAQ model state at that time step is used as $x_{b}$. After assimilation using 3DVAR within GSI, CMAQ returns to a free-running mode, and the new model state, x, is updated to more closely match the satellite observation. The difference in the monthly average NO_2_ VCDs from the assimilation and no-assimilation runs is used in the inversion as ΔΩ.

### 2.4 Finite-difference mass-balance inversion

**Figure 3.**
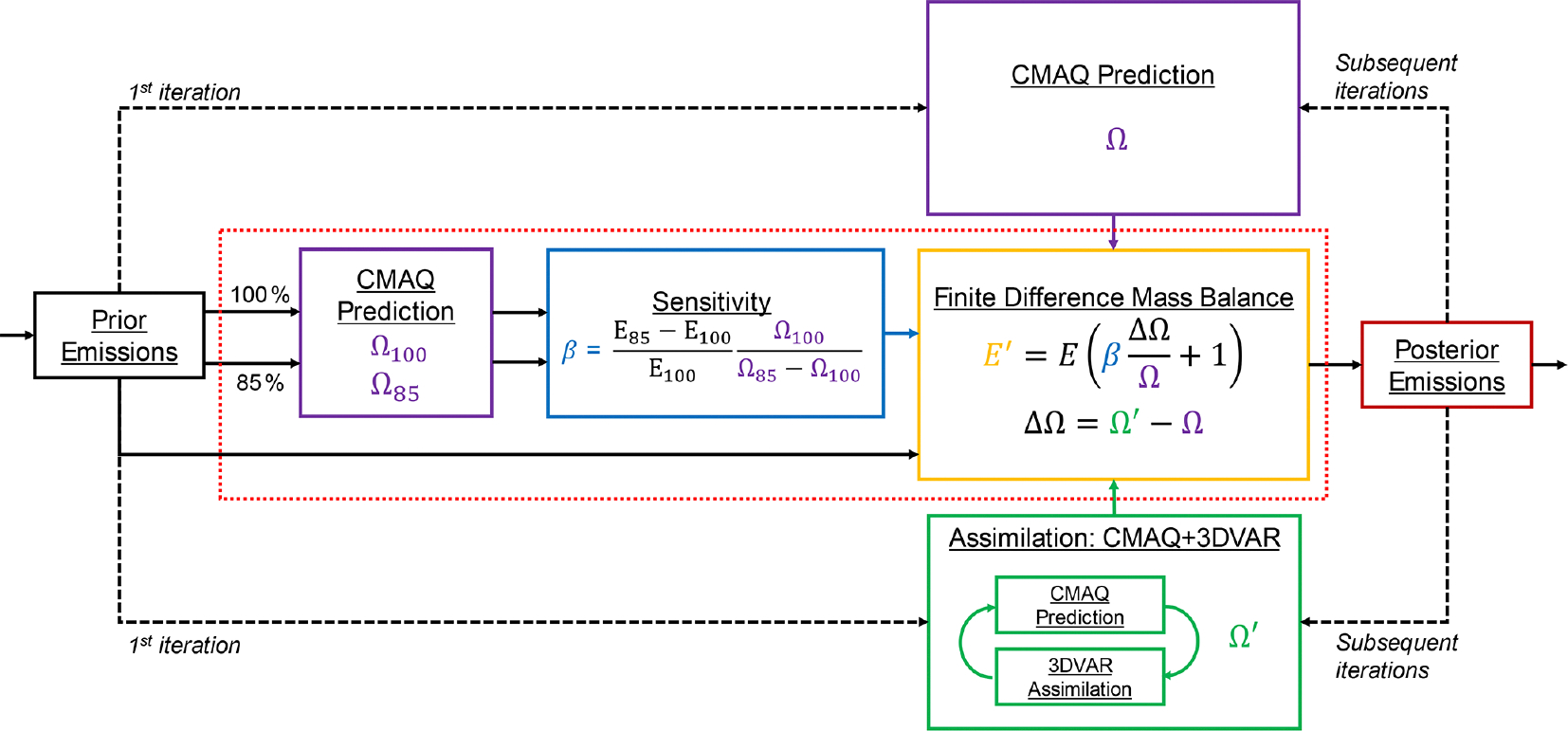
FDMB inversion. The red dashed line corresponds to the red dashed lines in Fig. 1, and the processes inside show additional details of the FDMB inversion. In this framework, the prior emissions (black box on the far left) are input to the CMAQ model. CMAQ simulations are performed with unperturbed prior emissions (100 % arrow and $E_{100}$) and prior emissions with a −15 % perturbation (85 % arrow and $E_{85}$). The resulting modeled VCDs are $\Omega_{100}$ and $\Omega_{85}$, respectively. These VCDs are used to compute the sensitivity, β (blue box). New emissions totals are calculated with FDMB (yellow box), using β, NO_2_ VCD from a CMAQ simulation without assimilation (Ω), and NO_2_ VCD from a CMAQ simulation with assimilation ($\Omega^{\prime }$). When iteration is used, the posterior emissions from the previous iteration are used as input to the CMAQ model to simulate new VCDs, Ω and $\Omega^{\prime }$.

In the inversion framework developed, we iterate the approach of Lamsal et al. (2011). The FDMB process as applied here is summarized in Fig. 3. In the past, this approach has been used by directly comparing model and satellite columns (e.g., Itahashi et al., 2019; Cooper et al., 2017; Lamsal et al., 2011). We modify the approach by first updating model concentrations with assimilation of satellite observations and then updating the emissions using the difference between the modeled VCD with and without assimilated satellite information. All updates are performed on a monthly average basis.

In FDMB, following Lamsal et al. (2011), emissions changes are inferred through the relationship

(5) $$\frac{\Delta E}{E}=\beta \frac{\Delta \Omega }{\Omega },$$

where ΔE is the inferred NO_*x*_ emissions change, E is the NO_*x*_ emissions prior, Ω is the model simulated NO_2_ VCD without chemical data assimilation, and $\Delta \Omega =\Omega_{assim}-\Omega$ is the monthly average difference between the model simulated tropospheric NO_2_ VCD with $\left(\Omega_{\text{assim}}\right)$ and without (Ω) chemical data assimilation. β is a unitless scaling parameter, the Jacobian, that linearly relates NO_2_ VCD changes to NO_*x*_ emissions changes. β is calculated through finite differencing as

(6) $$\beta =\frac{E^{\prime }-E}{E}\frac{\Omega_{E}}{\Omega_{E^{\prime }}-\Omega_{E}},$$

where $E^{\prime }$ is perturbed NO_*x*_ emissions, $\Omega_{E}$ is the tropospheric NO_2_ VCD simulated with model emissions E, and $\Omega_{E^{\prime }}$ is the tropospheric NO_2_ VCD simulated with model emissions $E^{\prime }$. To estimate β, we use the same −15 % perturbation used to create background errors B in the boundary layer. Cooper et al. (2017) found that using perturbations ranging from 5 % to 20 % to calculate β changed posterior emissions estimates by less than 2 % globally.

### 2.5 Inverse modeling NO_*x*_ emissions

In our framework, LNO_*x*_ emissions are updated first, separately from anthropogenic emissions. Due to the satellite instruments’ sensitivity to NO_2_ in the upper atmosphere (e.g., Eskes and Boersma, 2003), small model biases there can influence the total column comparison and adversely impact the anthropogenic emissions adjustment. By updating LNO_*x*_ emissions, we aim to decrease this bias and its impact on the ANO_*x*_ inversion. We compute the scaling parameter for lightning emissions, $\beta_{{LNO}_{x}}$, using the −15 % LNO_*x*_ perturbation simulations applied to create background errors for the upper troposphere. We then assimilate satellite NO_2_ observations using the background errors for the upper troposphere and apply $\beta_{{LNO}_{x}}$ in a single inversion iteration using the full tropospheric VCD to compute spatially varying LNO_*x*_ adjustment factors. Updates to LNO_*x*_ are calculated using monthly averages.

After LNO_*x*_ emissions are updated, ANO_*x*_ emissions are updated by iteratively applying an FDMB inversion independently for each month in 2019. Iterating the FDMB has been shown to improve emissions estimates compared to a single FDMB application (Cooper et al., 2017). In the FDMB iteration, each update to the emissions serves as the prior emissions for the subsequent iteration (represented as black dashed lines in Fig. 1). The number of iterations is determined based on the synthetic observation experiment described in Sect. 2.6. β is held constant during all ANO_*x*_ inversion iterations and is not recalculated each time to prevent instability in β as changes in the column become smaller with subsequent iterations. In the ANO_*x*_ emissions inversion, we only consider grid cells in which local anthropogenic NO_*x*_ emissions likely contribute significantly to the satellite-observed NO_2_ column by only including grid cells in which anthropogenic NO_*x*_ emissions comprise at least 50 % of total NO_*x*_ emissions, following Lamsal et al. (2011); population density is greater than 15000 peoplekm^−2^ (CIESIN, 2018); modeled cloud cover is less than 30 %; and the local time is 13:00 or 14:00 (OMI and TROPOMI overpass times). Only emissions in grid cells meeting these criteria are adjusted. Table S1 in the Supplement describes each simulation performed for the LNO_*x*_ and ANO_*x*_ inversions.

The FDMB method assumes that emissions impacts are local (i.e., emissions in one grid cell do not affect VCD amounts in neighboring grid cells). This assumption is most valid when NO_*x*_ lifetime is shorter than NO_*x*_ transport time to neighboring grid cells, which is typical near the surface in coarse-resolution models (Martin et al., 2003), such as the one used in this study. However, the assumption is less realistic at finer resolutions and in the upper troposphere, where the lifetime of NO_2_ is longer than at the surface and where NO_2_ concentrations are not directly impacted by coincident near-surface emissions. Even at coarse resolutions (e.g., 100 km grid spacing), emissions-smearing effects, which occur when the FDMB assumption of local emissions effects is incorrect and emissions are inappropriately adjusted, can appear due to NO_*x*_ transport, reservoir species, and chemical feedbacks (Turner et al., 2012; Cooper et al., 2017). Traditional FDMB, which directly compares modeled and remotely sensed columns, cannot address this effect. Assimilating the satellite VCD introduces an additional complication. The horizontal length scales (on the order of several hundreds of kilometers) used in the background error extend beyond the grid cell horizontal dimensions (nominally 108 km) in the middle and upper troposphere; as a result, NO_2_ changes introduced by assimilation (ΔΩ) do not have a local relationship with surface emissions directly below. In our work, assimilating the observed column information instead of directly comparing modeled and satellite-retrieved VCDs allows the analysis to be restricted to the lower troposphere, mitigating both the misallocation errors of FDMB and the effect of horizontal length scales extending beyond the grid cell dimension. To that end, we limit the anthropogenic emissions analysis to the lowest 20 model layers, which is nominal from the ground to ~ 720hPa over non-mountainous terrain in the summer, and use that partial column to calculate ΔΩ in the FDMB inversion. ΔΩ for a single month above and below the threshold is illustrated in Fig. S1 in the Supplement. By applying this cutoff, we focus the inversion on surface anthropogenic NO_*x*_.

### 2.6 Inversion system testing

We conduct a synthetic observation experiment to evaluate the ability of the inversion system to constrain emissions to a known perturbation. Artificial NO_2_ observations were generated from CMAQ simulations with unperturbed emissions and NO_*x*_ emissions reduced by 15 %. As expected, assimilating the synthetic observations derived from a simulation with unperturbed emissions results in an analysis increment of zero. The results of an iterative emissions inversion based on the synthetic observations derived from the simulation with perturbed emissions are shown in Fig. S2. Across Northern Hemisphere regions, the normalized mean error (NME) relative to the known perturbed emissions and the rate at which it changes decrease with subsequent iterations. The NME is minimized after seven to nine iterations, depending on the region. In all subsequent results, emissions inferences made with eight iterations of the inversion system are shown and analyzed. Convergence of the inversion in different global regions adds confidence to the system’s ability to constrain real-world emissions.

## 3 Results

### 3.1 Lightning NO_*x*_ emissions updates

Assimilation of retrievals from either satellite increases LNO_*x*_ emissions across all seasons, relative to the prior emissions (monthly climatology from GEIA), with the largest changes occurring during the summer (Figs. S3 and S4). Applying 2019 OMI data increases total LNO_*x*_ emissions in 2019 by 20 % over the GEIA climatology, while assimilation of TROPOMI data increases LNO_*x*_ emissions by 24 %. The emissions increases inferred by both satellite products are driven by NO_2_ increases in the mid and upper troposphere due to assimilation, with changes near the surface being negligible in comparison. Increases in background areas with small NO_2_ column totals and subsequent LNO_*x*_ increases in these areas suggest a low bias in modeled background NO_2_ relative to observations from both satellites. A low bias agrees with the findings reported by other model and satellite NO_2_ comparisons (Silvern et al., 2019; Qu et al., 2021; Goldberg et al., 2017). The LNO_*x*_ emissions adjustments inferred here decrease the differences between modeled and satellite-derived NO_2_ in the upper troposphere and decrease the bias that differences in the upper troposphere can introduce to the subsequent ANO_*x*_ inversion.

### 3.2 Impact of assimilation on modeled NO_2_ vertical column density

**Figure 4.**
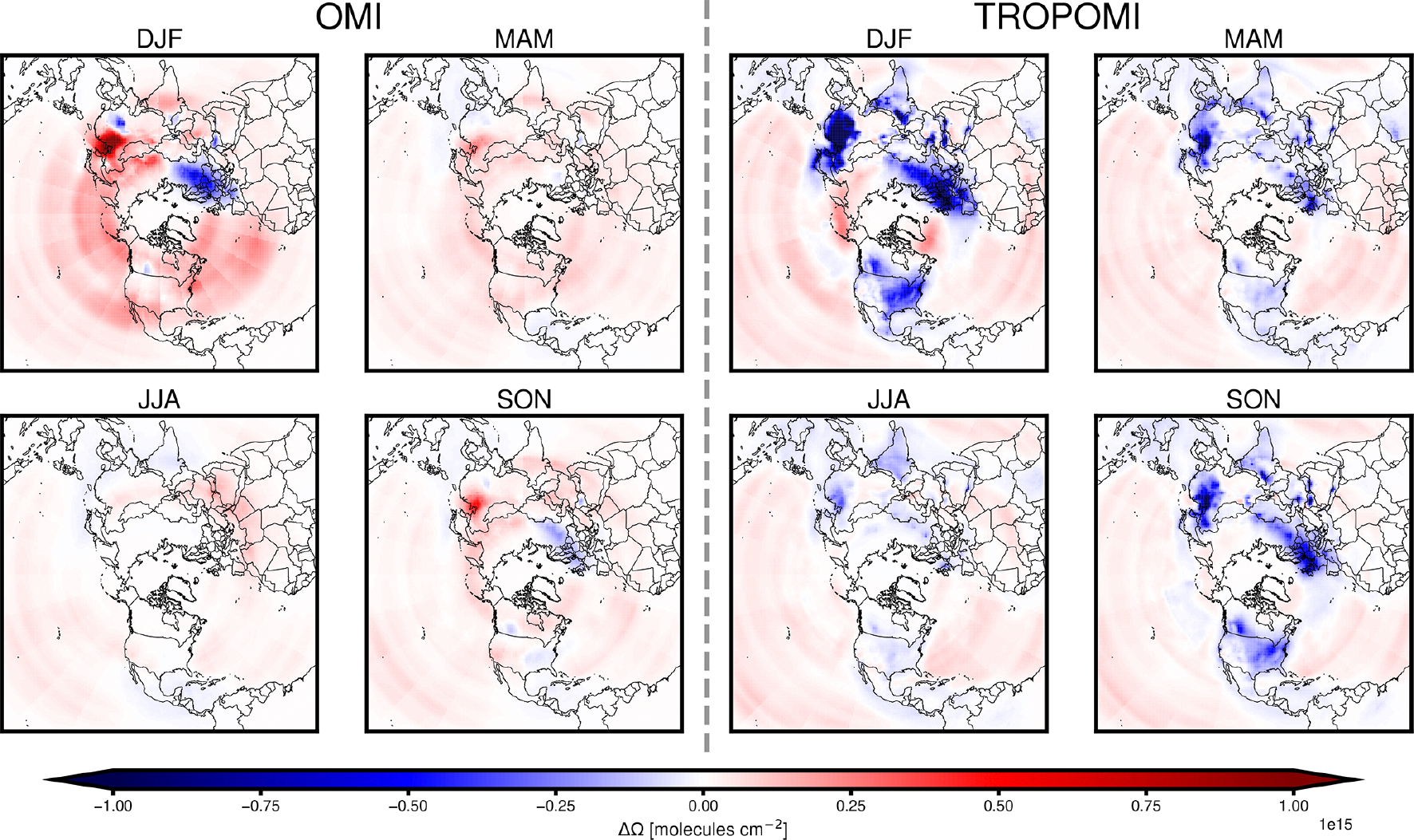
Seasonal NO_2_ VCD change (ΔΩ) from CMAQ simulation using prior emissions after assimilating OMI or TROPOMI tropospheric NO_2_ observations and modeling atmospheric composition with prior NO_*x*_ emissions. ΔΩ shown for winter (DJF), spring (MAM), summer (JJA), and fall (SON).

Figure 4 shows the change to CMAQ-modeled tropospheric VCD, (ΔΩ) caused by assimilating NO_2_ observations from OMI or TROPOMI with background errors for the boundary layer, before applying any emissions adjustments. In Fig. 4 and throughout the results, ΔΩ reflects differences near the surface (as described in Sect. 2.5). Assimilating OMI NO_2_ data generally increases modeled NO_2_ columns near populated areas in China, India, and the US. In contrast, assimilating TROPOMI NO_2_ data decreases modeled NO_2_ columns more widely across the Northern Hemisphere. The changes brought about by assimilating satellite data are larger during the winter and fall and smaller in the spring and summer, when NO_*x*_ lifetime is shortest and when NO_2_ columns are smaller. During the winter in northeast China, where the assimilation impacts are most apparent, the seasonal average change due to assimilation reaches 1.8 × 10^15^ molec.cm^−2^ for OMI and −2.8 × 10^15^ molec.cm^−2^ for TROPOMI. The direction of ΔΩ after assimilation of OMI data is more heterogeneous and shows a stronger seasonality, while ΔΩ based on assimilating TROPOMI data is consistently negative. Over Europe, ΔΩ after assimilating OMI observations is close to zero in warm months and negative in colder seasons. Assimilating satellite-observed NO_2_ increases the NO_2_ levels modeled over the ocean and less-populous areas, such as the Sahara, with low NO_*x*_ emissions and small NO_2_ column amounts.

Over polluted areas, the direction of ΔΩ for the TROPOMI or OMI data assimilations tends to differ. This discrepancy is likely due to the low bias in TROPOMI-derived tropospheric NO_2_ columns, which has been reported to be approximately 10 % over the US, Europe, and India, and greater than 20 % over China when compared with the OMI Quality Assurance for Essential Climate Variables (QA4ECV) retrieval (Van Geffen et al., 2022; Verhoelst et al., 2021; C. J. Wang et al., 2020; Li et al., 2021). Over background areas, the analysis increments that result from assimilation of observations from both satellites generally agree. The consistency suggests a low bias in modeled background NO_2_ concentrations and also agrees with the low bias in CMAQ-modeled free-tropospheric NO_2_ reported by Goldberg et al. (2017). Such a bias can contribute to the positive analysis increment over background areas. However, NO_2_ columns observed in these regions may be smaller than the retrieval accuracy of 0.7 × 10^15^ molec.cm^−2^ (Van Geffen et al., 2019), reducing confidence in the analysis increment at these locations. In the anthropogenic emissions inversion, our filtering criteria exclude background areas which are more likely to have low VCD amounts.

### 3.3 Emissions inversion

Season-average β values, relating NO_2_ vertical column differences to anthropogenic near-surface NO_*x*_ emissions updates, are shown in Fig. S5. Based on our criteria for grid cell inclusion in the inversion, described in Sect. 2.5, we consider 13 % of the grid cells in the domain, which represent 88 % of prior anthropogenic NO_*x*_ emissions. Seasonal domain-average values range from 1.33 to 1.66 and are lower in the winter and higher in the summer. A β value less than 1.0 results in an emissions update that is smaller than the VCD change, while a β greater than 1.0 has the opposite effect. β tends to be less than 1.0 in polluted regions during colder months and larger during warmer months and in less-polluted regions, although many grid cells which are less polluted are not considered in the analysis. The scaling factors are smallest over China and larger over the US, India, Mexico, and Europe. The differences among regions stem from local differences in NO_*x*_ lifetime and transport. In Indonesia and sub-Saharan Africa, lower emissions and a small response from tropospheric VCD to anthropogenic emissions perturbations can lead to large β values. To prevent overly large or small β values, we constrain the factor to between 0.1 and 10, following Cooper et al. (2017). Scaling factors estimated here are larger than the 1.16 global-average previously reported by Lamsal et al. (2011). However, in Lamsal et al. (2011), modeled NO_2_ vertical columns were sampled at the morning SCanning Imaging Absorption spectroMeter for Atmospheric CHartographY (SCIAMACHY) overpass time rather than at the afternoon OMI or TROPOMI overpass times; β tended to be closer to 1.0 during the morning in regions with high NO_*x*_ emissions (Li and Wang, 2019). Li and Wang (2019) show that, over rural regions with lower NO_*x*_ concentrations, β is larger at the OMI or TROPOMI overpass window than at the SCIAMACHY overpass window, suggesting that a larger overall β for analyses based on OMI or TROPOMI products should be expected. Additionally, NO_*x*_ emissions have decreased considerably in several regions of the Northern Hemisphere, including the US (Tong et al., 2015) and China (Miyazaki et al., 2017), after the Lamsal (2011) study was conducted, which has changed the sensitivity of NO_2_ VCDs to NO_*x*_ emissions (Qu et al., 2021; Silvern et al., 2019).

**Figure 5.**
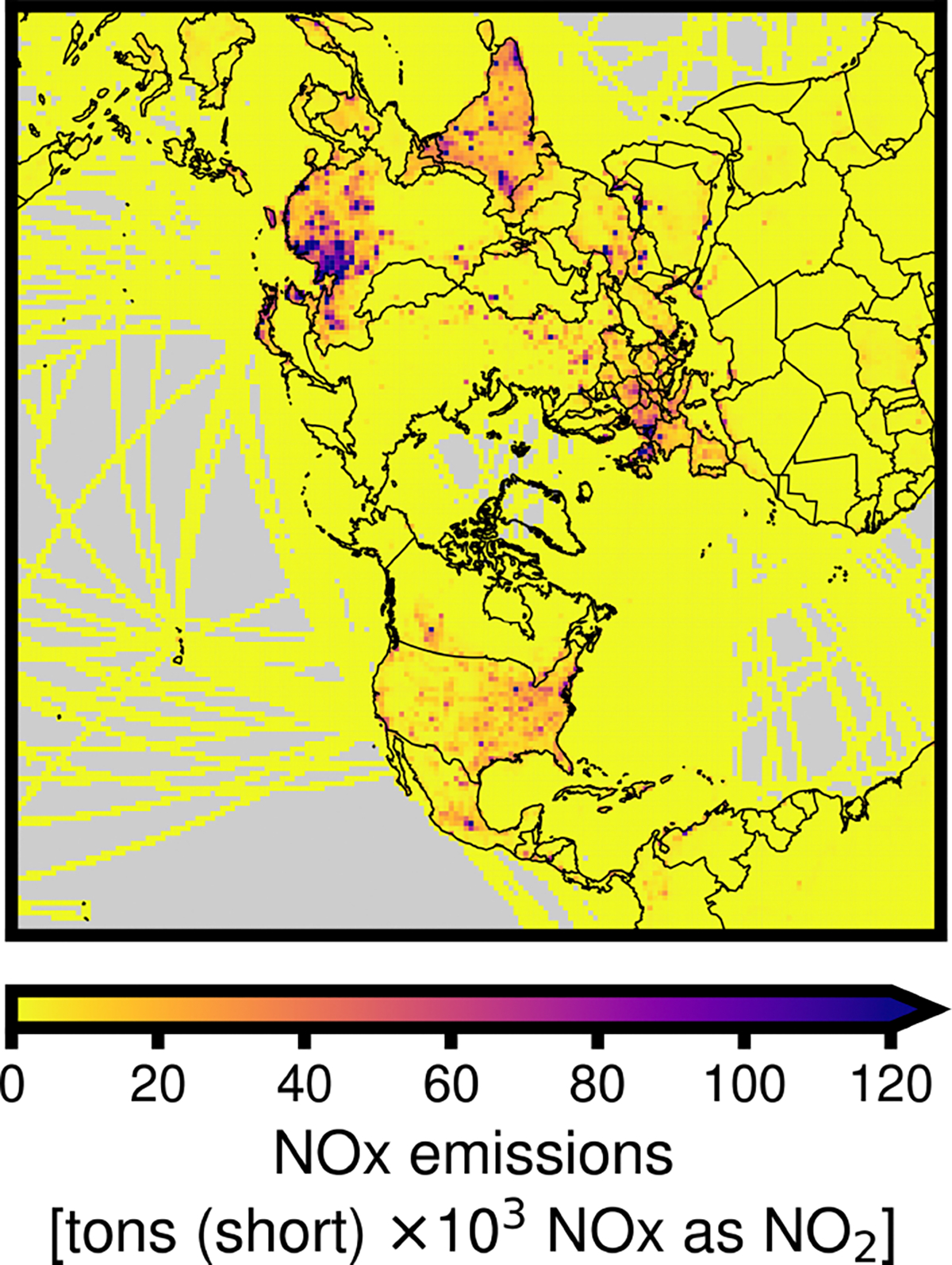
2019 prior anthropogenic NO_*x*_ emissions totals. Data sources are described in Table 1.

**Figure 6.**
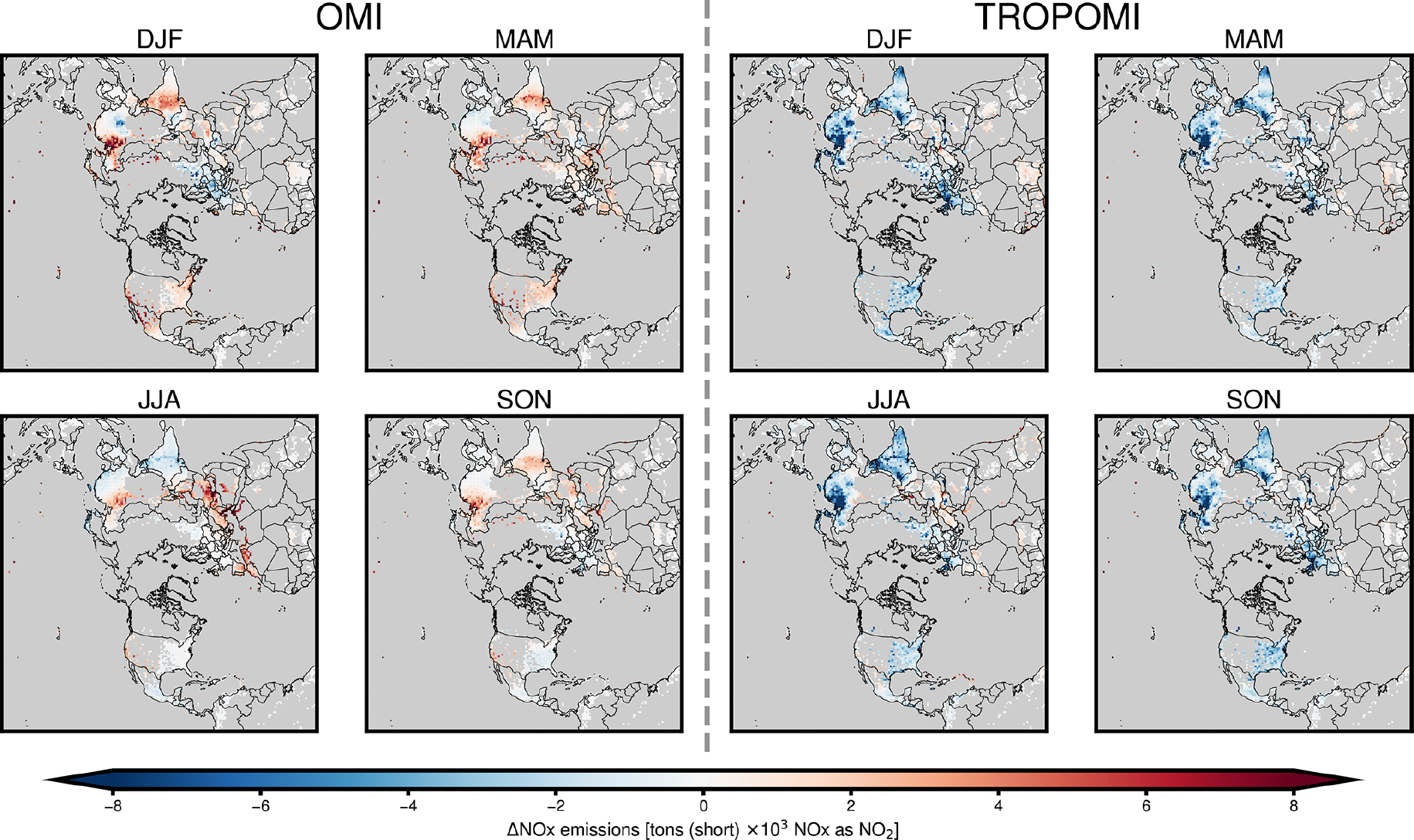
Season-average NO_*x*_ emissions changes from inverse modeling updates based on OMI and TROPOMI observations. Emissions changes are shown for winter (DJF), spring (MAM), summer (JJA), and fall (SON).

Annual bottom-up prior ANO_*x*_ emissions estimates are shown in Fig. 5. Season-average ANO_*x*_ emissions inferences from the inversions based on OMI and TROPOMI observations are shown in Fig. 6. The use of OMI observations generally tends to increase emissions in most industrialized nations outside of Europe. NO_*x*_ emissions increases driven by OMI observations are largest in winter and spring and smaller, or slightly decreased, in summer and fall. In contrast, the use of TROPOMI retrievals tends to drive a decrease in NO_*x*_ emissions across all seasons and continents, with the largest impacts in the summer and the smallest in the spring. The largest emissions changes based on both OMI and TROPOMI retrievals are in northeast China during the winter. Over India, OMI-inferred changes are concentrated in central India, where prior emissions are lower, while the largest changes inferred from TROPOMI are in the northern, eastern, and southern zones, where prior emissions are highest. Relative NO_*x*_ emissions changes driven by TROPOMI observations tend to be small over dense urban areas, with more uniform decreases over cells with lower emissions.

**Figure 7.**
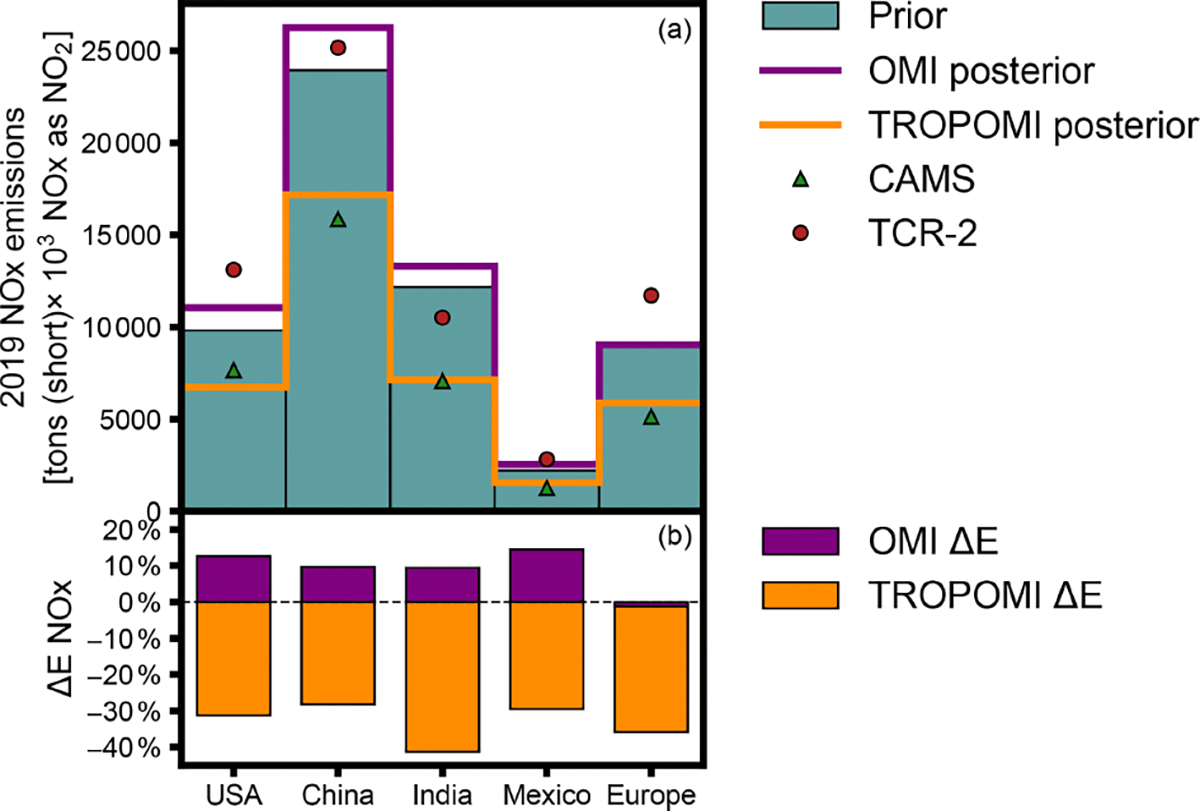
Prior and satellite-inferred 2019 anthropogenic NO_*x*_ emissions in select global regions. Plot (**a**) shows total emissions (as NO_2_) from prior emissions estimates, inference with OMI or TROPOMI observations (OMI and TROPOMI posterior), and CAMS or TCR-2 inventories in the US, China, India, Mexico, and Europe. Plot (**b**) shows the percent change (Δ*E*NO_*x*_) inferred with OMI or TROPOMI data, relative to prior emissions estimates, for each region.

ANOx emissions totals and inferred changes are explored for China, India, Europe, Mexico, and the US (Fig. 7). We also show 2019 NO_*x*_ emissions totals from the Copernicus Atmosphere Monitoring Service’s (CAMS’s) bottom-up emissions inventory (Granier et al., 2019) and from the NASA Tropospheric Chemical Reanalysis products 2 (TCR-2) satellite-inferred inventory (Miyazaki et al., 2019, 2020). TCR-2 top-down NO_*x*_ emissions are constrained using satellite observations of NO_2_, CO, O_3_, and SO_2_ at a resolution of 1.125° × 1.125° and are further described in Miyazaki et al. (2017). CAMS anthropogenic NO_*x*_ emissions are based on the Emissions Database for Global Atmospheric Research (EDGAR version 5.3) estimates for 2015 (Crippa et al., 2020), projected to 2019 using CEDS scaling factors, and are provided at 0.1° × 0.1°. Both datasets provide monthly anthropogenic NO_*x*_ totals. Except for Europe, assimilation toward OMI retrievals increases annual emissions totals in the regions analyzed, while using TROPOMI retrievals decreases them. TCR-2 NO_*x*_ emissions estimates are larger than the prior emissions used by our inverse modeling framework, except for India, while CAMS totals are lower than the prior emissions estimates and are similar to TROPOMI inferred emissions. Across the regions considered, TROPOMI infers an average annual decrease of −33 % in NO_*x*_ emissions from the regions, while OMI infers a +9 % increase. In Europe, the only region where the sign of the inferred changes match, the use of OMI retrievals results in a −1 % change, while applying TROPOMI observations leads to a −36 % decrease in NO_*x*_ emissions. The largest total changes are inferred in the highest-emitting region, China, while the greatest relative changes, −41 % inferred with TROPOMI, are for India, where emissions are highly uncertain. Changes inferred with OMI observations over the US are greater than 1200 × 10^3^ short tons NO_*x*_ as NO_2_ per year but smaller than the difference between our prior US emissions estimates and TCR-2 or CAMS estimates. A change of −3000 × 10^3^ short tons NO_*x*_ as NO_2_ emitted annually in the US, as inferred by TROPOMI, over 30 % of the prior emissions differs significantly from National Emissions Inventory estimates but leads to a total close to that of the 2019 CAMS inventory.

**Figure 8.**
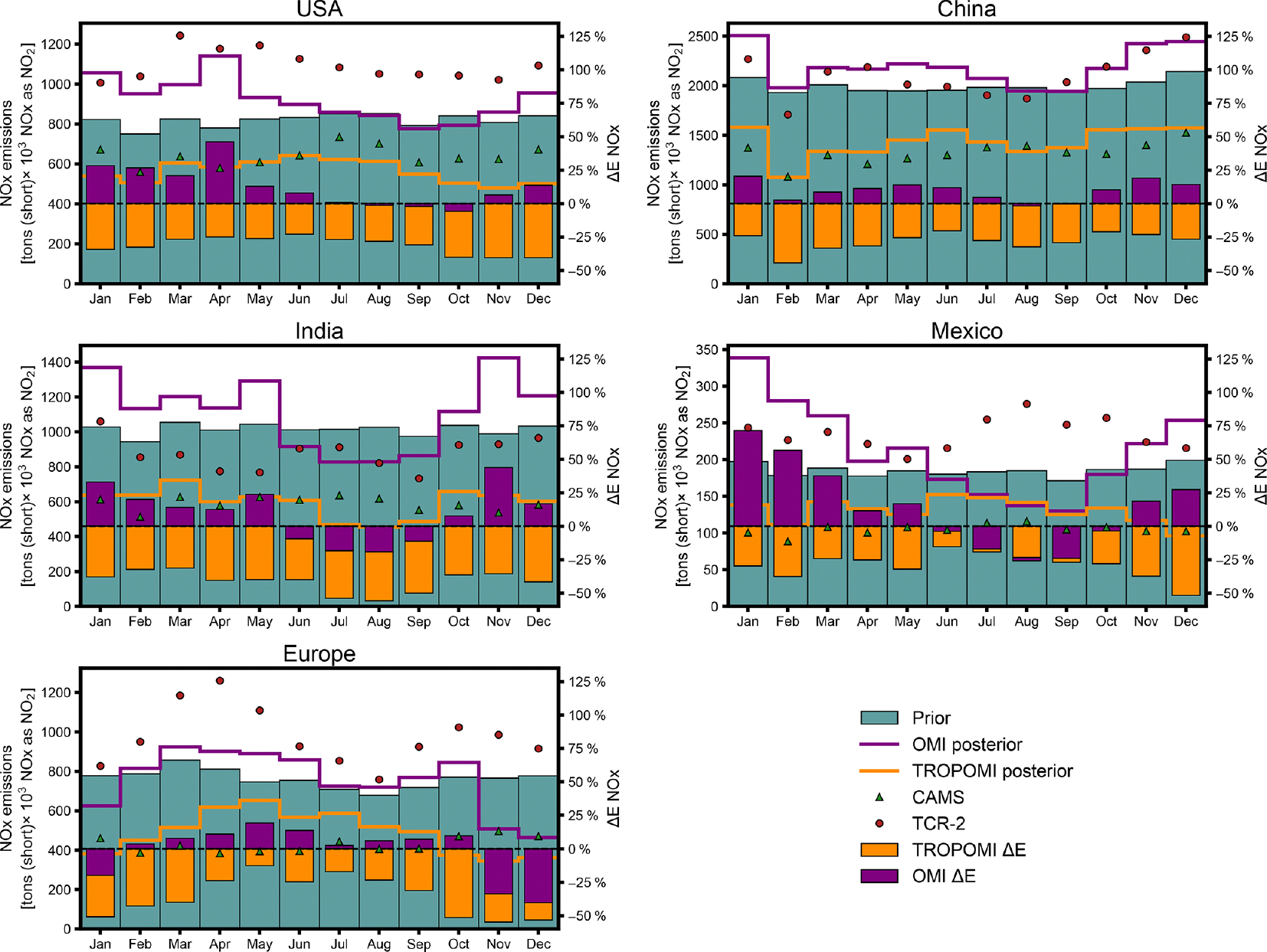
Monthly prior and satellite-inferred anthropogenic NO_*x*_ emissions in 2019 in select global regions. Total monthly emissions (as NO_2_) from prior emissions estimates, inference with OMI or TROPOMI observations (OMI and TROPOMI posterior), and CAMS or TCR-2 inventories in the US, China, India, Mexico, and Europe are shown. Percent changes (Δ*E*NO_*x*_) inferred with OMI or TROPOMI data, relative to prior emissions estimates, for each region are shown by the purple and orange bars.

Across the months simulated, inferences using OMI retrievals consistently lead to higher NO_*x*_ emissions than using TROPOMI retrievals. Figure 8 shows monthly NO_*x*_ emissions totals and inferred changes for several global regions. The magnitude of changes is generally smallest in summer months and largest in winter months for both OMI- and TROPOMI-inferred emissions. Monthly prior emissions totals lay between the OMI and TROPOMI inferences, except for summertime emissions in India and Mexico, where both satellite inferences decrease NO_*x*_ emissions. Over Europe, both satellite products infer a decrease during the winter, although fewer valid satellite pixels due to snow cover at high latitudes and longer winter NO_2_ atmospheric lifetimes may influence the inference.

Based on reported NO_*x*_ emissions trends (McDuffie et al., 2020; U.S. EPA, 2022a), changes from the prior emissions inventory (Table 1) to 2019 are expected. Relative to the prior emissions, significant decreases in NO_*x*_ emissions in China, estimated for 2015 in the prior inventory, and smaller reductions in Europe and North America, reported for 2014 and 2017 in the prior inventory, respectively, should be anticipated. TROPOMI-inferred emissions reflect the direction anticipated for these changes but with larger than expected magnitudes. For example, the 28 % decrease in anthropogenic NO_*x*_ emissions over China between 2015 and 2019 inferred from the TROPOMI observations is substantially larger than the 8 % decrease estimated between 2015 and 2017 by the Community Emissions Data System (McDuffie et al., 2020). Bottom-up estimates indicate that anthropogenic NO_*x*_ emissions in the US have decreased through 2019 (U.S. EPA, 2022a). Although the direction of the emissions change inferred from TROPOMI agrees with the trend in bottom-up estimates, its magnitude is larger than expected. An underestimation of US emissions in winter in the prior inventory when compared with OMI inferences contrasts with field study results reporting no bias in northeastern US winter emissions estimates (Jaegle et al., 2018; Salmon et al., 2018). In India, bottom-up emissions inventories report sustained growth of NO_*x*_ emissions (Kurokawa and Ohara, 2020; McDuffie et al., 2020), and NO_2_ levels observed by OMI have been increasing since 2005 (Goldberg et al., 2021; Cooper et al., 2022). The decrease in anthropogenic NO_*x*_ emissions inferred by TROPOMI observations contrasts with these trends in bottom-up estimates and OMI observations.

The low bias known to affect TROPOMI NO_2_ observations influences the results of the emissions inversion, which targets grid cells with high emissions, likely leading to decreases in inferred emissions that are larger than expected. We conduct an inversion using the reprocessed TROPOMI NO_2_ version 2.3.1 (Van Geffen et al., 2022) to infer NO_*x*_ emissions for January 2019 and find that the updated data increase the TROPOMI posterior inference by 17 % over the US and 4 % in China relative to version 1.2.2. While using the updated retrievals shrinks the gap between OMI- and TROPOMI-inferred emissions, it does not change the overall trend of smaller posterior emissions using TROPOMI NO_2_ (Figs. S10 and S11). The differences between emissions inferred by OMI and TROPOMI observations highlight the importance of ongoing efforts to harmonize OMI and TROPOMI NO_2_ retrieval algorithms, such as the NASA Multi-Decadal Nitrogen Dioxide and Derived Products from Satellites (MINDS) (Lamsal et al., 2020) and the QA4ECV (Boersma et al., 2017) datasets.

In addition to smearing effects, coarse-resolution models can artificially alter nonlinear NO_2_ chemistry, leading to biases in inferences of NO_*x*_ emissions from satellite NO_2_ columns (Valin et al., 2011; Sekiya et al., 2021; Lamsal et al., 2011). Higher-resolution simulations can better resolve β and reduce biases caused by nonlinear chemistry. Additional errors in the emissions estimates may be associated with emissions from non-anthropogenic NO_*x*_ sources. Although the emissions inversion targets anthropogenic sources only, changes in NO_2_ columns observed by the satellite instruments driven by natural NO_*x*_ emissions processes may not be captured in the air quality model simulations and subsequently lead to biased anthropogenic emissions inferences (Li et al., 2021).

The emissions resulting from the inverse modeling framework are comparable to CAMS and TCR-2 2019 emissions estimates in several ways. In the US, China, and Europe, the magnitudes of ANO_*x*_ emissions from OMI retrievals are comparable to TCR-2 NO_*x*_ emissions estimates and exhibit similar monthly patterns. Annual NO_*x*_ emissions inferred from OMI observations are also relatively similar to TCR-2 estimates for India and Mexico, although monthly emissions patterns differ. Unlike TCR-2 emissions estimates, which are also constrained by OMI NO_2_ observations, 2019 CAMS emissions estimates are projected from 2015 bottom-up data. However, CAMS estimates provide a representation of anticipated emissions trends. In all regions considered, CAMS NO_*x*_ emissions estimates are close to the TROPOMI inference annual totals and lower than the prior emissions, OMI inferences, and TCR-2 estimates, potentially suggesting that global NO_*x*_ emissions have not decreased as much as anticipated by the CAMS inventory projections.

### 3.4 Impacts of emissions updates on modeled NO_2_ and O_3_

**Figure 9.**
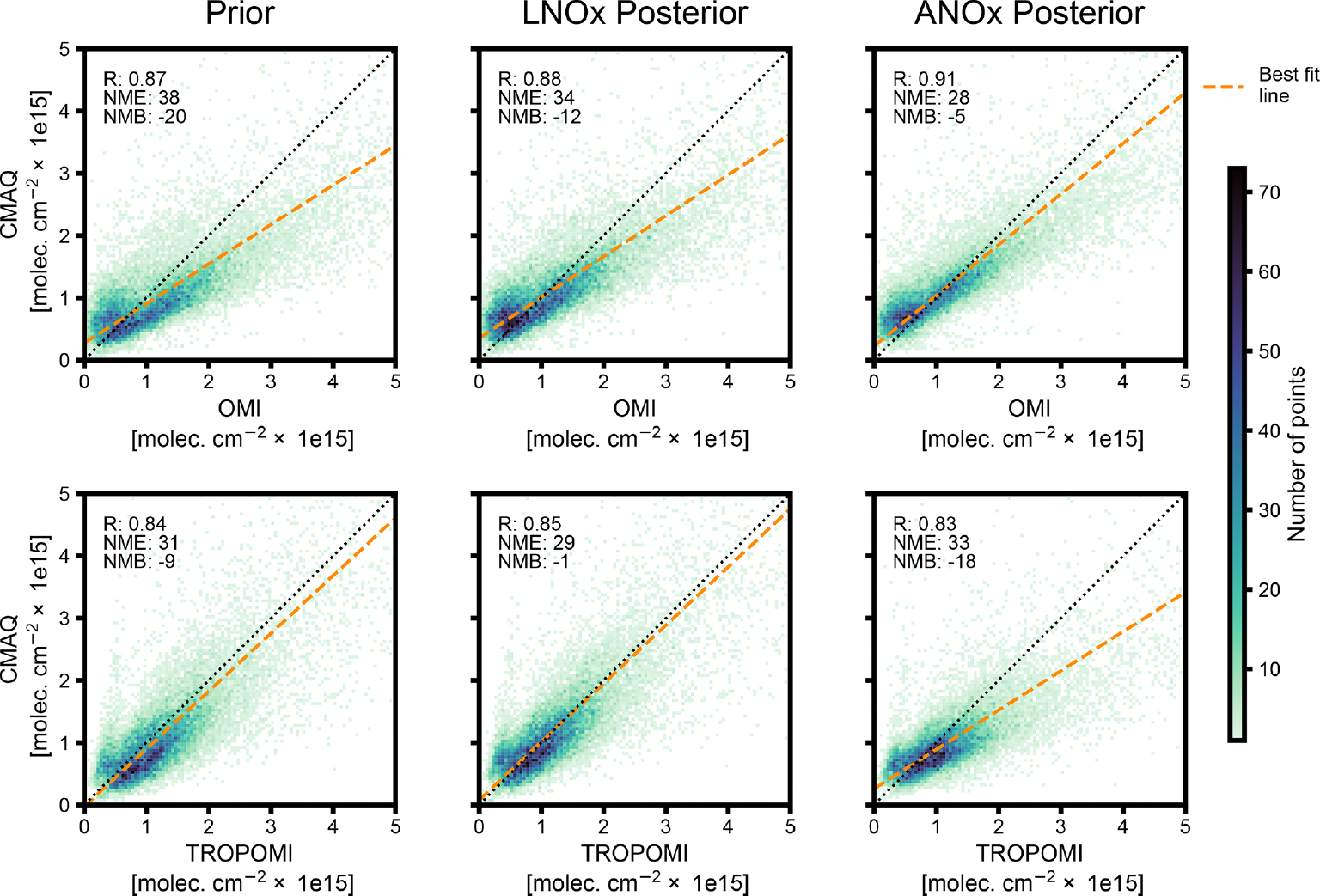
Impact of NO_*x*_ emissions updates on modeled NO_2_ VCDs. Plots compare 2019 season-average CMAQ-modeled NO_2_ VCD at each model grid cell in which NO_*x*_ emissions were updated by the inverse modeling framework against OMI and TROPOMI tropospheric NO_2_ VCD retrievals averaged in each model grid cell. Modeled NO_2_ VCD using prior emissions (Prior), inferred LNOx emissions (LNOx posterior), and inferred lightning and anthropogenic NO_*x*_ emissions (ANO_*x*_ posterior) are each compared with NO_2_ VCD retrievals. Top-row plots compare retrievals and modeled VCD based on OMI observations, while bottom-row plots compare retrievals and modeled VCD based on TROPOMI observations. Linear regression line, correlation coefficient (*R*), normalized mean error (NME), and normalized mean bias (NMB), relative to tropospheric NO_2_ VCD retrievals, are shown for each CMAQ simulation.

We evaluate and compare the CMAQ simulations’ ability to reproduce observed pollutant concentrations when driven with NO_*x*_ emissions estimates from the prior inventory and with those derived by the inverse modeling framework. Figure 9 compares 2019 OMI and TROPOMI NO_2_ VCD retrievals with modeled NO_2_ VCDs using the prior emissions with no updates, LNO_*x*_ emissions updates, and LNO_*x*_ and ANO_*x*_ emissions updates. Satellite-based LNO_*x*_ emissions updates improve CMAQ model performance – correlation coefficient (*R*), normalized mean error (NME), and normalized mean bias (NMB) – when evaluated against tropospheric VCD retrievals, relative to model performance with the prior emissions. OMI-inferred ANO_*x*_ emissions updates further improve CMAQ model performance evaluated against VCD retrievals, decreasing NMB from −20 % to −5 % and NME from 38 % to 28 %. Model performance is improved by using OMI data in the inverse modeling framework across all seasons (Figs. S6–S9). Although LNO_*x*_ emissions updates derived from TROPOMI observations improve model bias and error relative to the CMAQ simulation using prior emissions estimates, TROPOMI-inferred anthropogenic emissions do not, except during summer months (Figs. 9 and S6–S9). The lack of significant improvements in CMAQ-simulated NO_2_ VCDs after applying the emissions inversion with TROPOMI NO_2_ retrievals prior to the version 2.3.1 update (Van Geffen et al., 2022) may be associated with changing chemical regimes that are not captured in the emissions inversion process.

Changes in modeled VCD due to assimilation and the emissions inferences calculated in the TROPOMI ANO_*x*_ inversion exceed the emissions perturbation and VCD changes used to calculate β. For example, over the eastern US, the −15 % emissions perturbation used to calculate β leads to VCD changes of −15 % on average in winter, but assimilating TROPOMI retrievals leads to VCD changes (ΔΩ) of −19 % on average in the winter, with individual changes exceeding −30 %. Modeled NO_*x*_ chemistry and NO_2_ vertical profiles after assimilating TROPOMI retrievals may be different than those used in the calculation of β. As a result, assimilating TROPOMI retrievals in the ANO_*x*_ inversion may lead to modeled NO_2_ vertical profiles which are inconsistent with the precalculated β used in the FDMB relationship and to less reliable subsequent emissions inferences. In contrast, the magnitude of VCD changes due to assimilating OMI retrievals over the eastern US in winter is 8 %, well within the magnitude of the VCD changes used to precalculate β. This highlights the importance of applying a β sensitivity valid for the magnitude of anticipated emissions changes in FDMB inversions and the potential consequences of relying on satellite-derived retrievals with pre-existing biases in emissions inversions.

**Figure 10.**
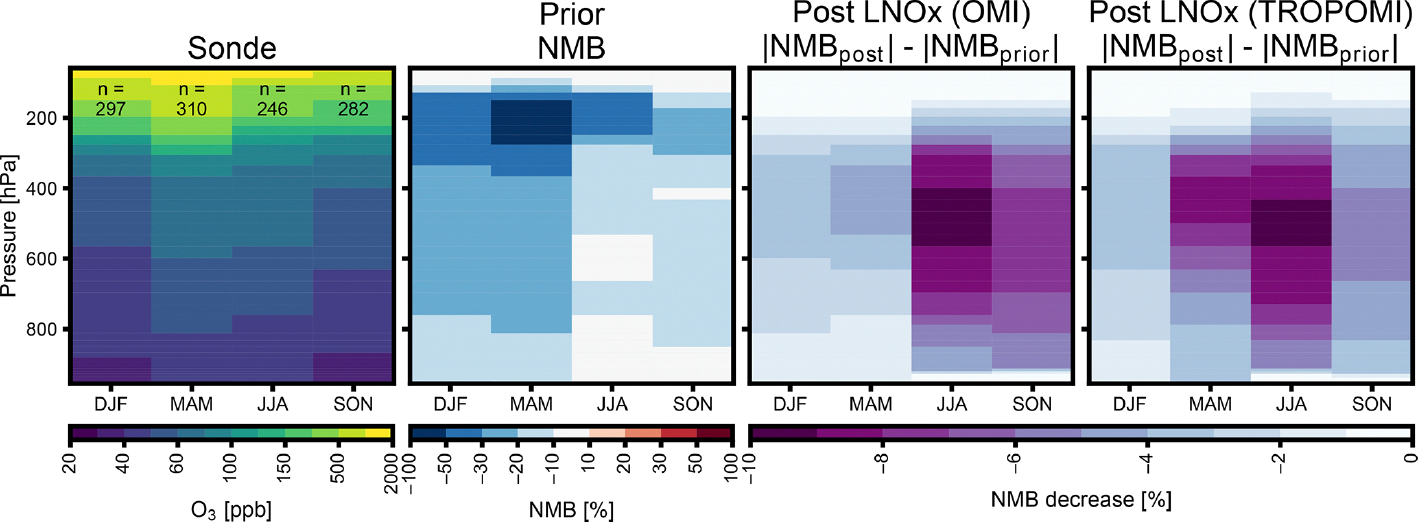
Ozonesonde observations from the WOUDC network and impact of lightning emissions inferences on modeled ozone. Left plot shows sonde observations averaged in each season and total number of launches per season. The NMB is shown for the prior emissions simulation. Plots on the right show the decrease in the NMB, relative to the prior simulation for simulations, with LNO_*x*_ emissions updated with OMI and TROPOMI data.

Comparing CMAQ-modeled O_3_ to ozonesonde measurements from the World Ozone and Ultraviolet Radiation Data Centre (WOUDC) network shows the impacts updating LNO_*x*_ emissions on simulated tropospheric O_3_ (Fig. 10). Above 300 hPa, the model is biased low, but neither update has a major impact on this bias. However, within the free troposphere, the effects of LNO_*x*_ emissions updates are larger. LNO_*x*_ satellite-inferred emissions from both satellites increase O_3_ and subsequently improve the model’s low O_3_ bias across all seasons, with the strongest effect in the summer. This suggests a low background NO_2_ in our prior simulation, consistent with several studies demonstrating that models underestimate background NO_2_ (Goldberg et al., 2017; Qu et al., 2021; Silvern et al., 2019).

Comparisons of CMAQ-modeled NO_2_ and O_3_ concentrations with ground-level measurements highlight the challenges of reproducing local air quality with a coarse scale model but suggest potential to improve model performance with satellite-derived NO_*x*_ emissions updates. Table 2 shows statistics evaluating modeled ground-level daily average NO_2_ and maximum 8 h O_3_ concentrations over the US against observations from 1218 monitoring sites in the Air Quality System (AQS) (U.S. EPA, 2022b), excluding near-road monitors for which the gridded NO_2_ fields are not representative. Statistics for each season are included in Tables S2 and S3. There is a significant low bias in CMAQ-predicted ground-level NO_2_ concentrations compared with monitoring site measurements, likely due to the model’s coarse grid resolution and the aggregation of NO_2_ monitors within urban areas with high NO_*x*_ emissions and large concentration gradients. CMAQ simulations at higher horizontal resolution do not show the same bias against NO_2_ surface observations (Toro et al., 2021). Agreement between modeled and observed ground-level NO_2_ concentrations is improved by using OMI-inferred NO_*x*_ emissions compared with the prior emissions simulation, particularly during winter and spring months. Model performance evaluated against ground-level O_3_ measurements improves to a smaller extent with OMI-inferred NO_*x*_ emissions during winter and spring months. The use of TROPOMI-inferred emissions has mixed impacts on CMAQ performance against observed ground-level NO_2_ and O_3_ concentrations, leading to limited gains in seasonal *R* and some seasonal biases and errors but also less agreement with observations for other seasonal statistics. In the US, the network of ground-based air quality observations is relatively large. However, in some regions where emissions uncertainties are expected to be especially high, ground-based observations are significantly limited and less accessible. Assessing the impact of emissions updated against ground-based observations in these regions, although a challenge, would provide further evaluation of the inversion framework in locations where satellite retrievals have the largest potential to provide important constraints to emissions estimates.

### 3.5 Impacts of emissions updates on long-range O_3_ transport

**Figure 11.**
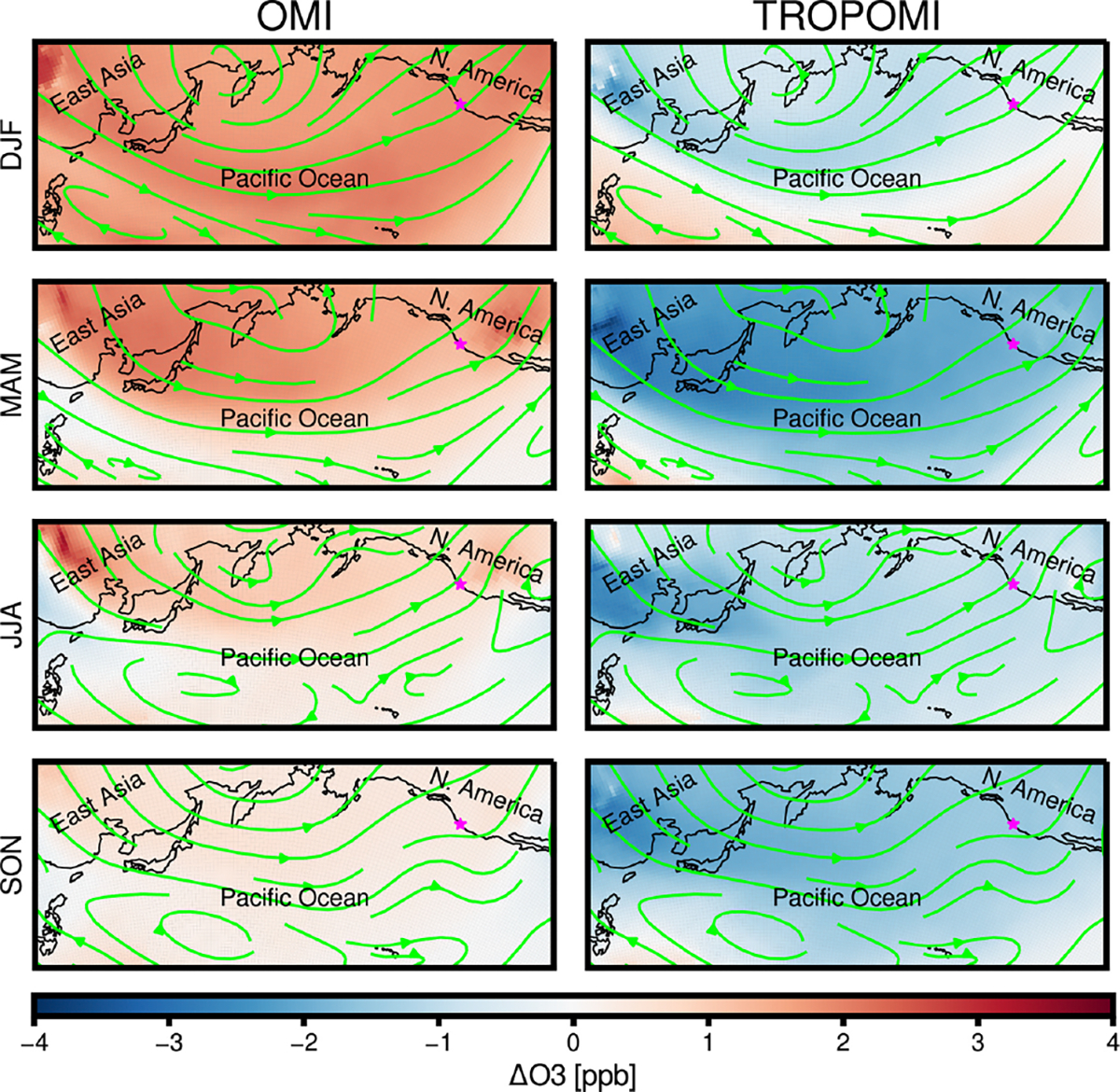
Changes in 2019 season-average free-tropospheric O_3_ concentrations (averaged between 750 and 250hPa) simulated over the North Pacific Ocean using lightning and anthropogenic NO_*x*_ emissions inferred with OMI or TROPOMI observations, relative to simulations using prior ANO_*x*_ emissions and updated LNO_*x*_ emissions. Differences are shown for winter (DJF), spring (MAM), summer (JJA), and fall (SON). Arrows depict season-average free-tropospheric winds (750–250hPa). Star marker indicates location of Trinidad Head, California.

Global NO_*x*_ emissions estimates affect model simulations of long-range air pollution transport. To explore these impacts, we examine the response of CMAQ-modeled trans-Pacific O_3_ to the inverse modeling framework’s NO_*x*_ emissions updates. Figure 11 shows season-average changes in simulated free-tropospheric O_3_ over the North Pacific Ocean resulting from the use of OMI- and TROPOMI-inferred ANO_*x*_ emissions relative to the emissions simulation with LNO_*x*_ emissions updated. As expected, the emissions inversions lead to O_3_ variations that follow NO_*x*_ emissions changes inferred for each satellite’s observations, with OMI inferences resulting in higher O_3_ concentrations and TROPOMI inferences resulting in lower O_3_ concentrations over the North Pacific Ocean. Season-average differences with respect to the prior emissions simulation are as large as +1.8ppb in winter, using OMI-based updates, and −1.9ppb in spring, using TROPOMI-based updates. Combined with trans-Pacific wind patterns, the effects of the NO_*x*_ emissions inversions on modeled O_3_ suggest potential implications of uncertain Asian emissions estimates for US air quality management and emphasize the impacts of biases in satellite retrievals on inverse modeling systems.

**Figure 12.**
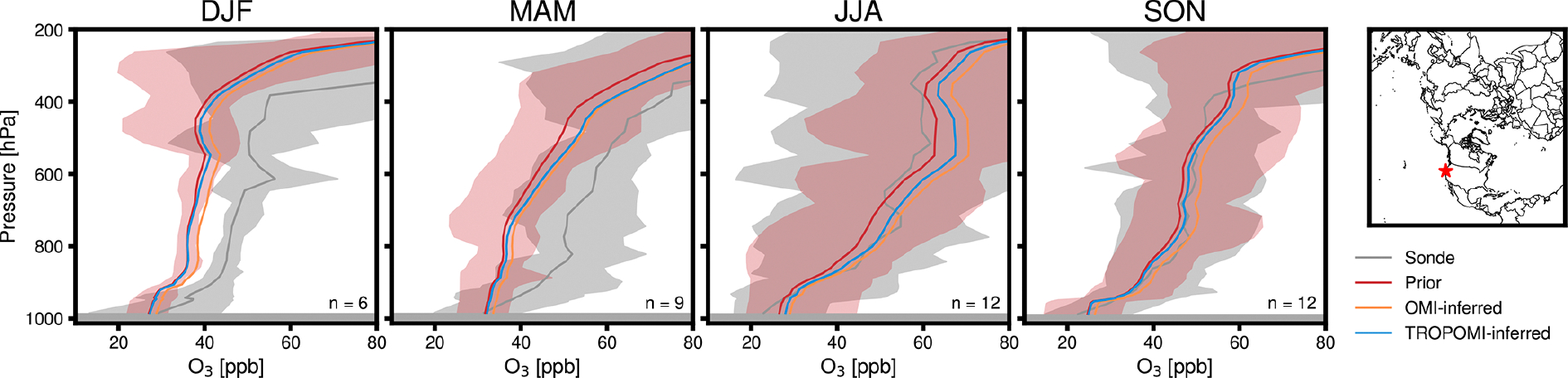
Season-average vertical O_3_ concentration profiles modeled by CMAQ and measured by ozonesondes launched at Trinidad Head, California, in 2019. Vertical distributions are shown for simulations using prior emissions (Prior), lightning and anthropogenic NO_*x*_ emissions inferred with OMI data (OMI-inferred), and lightning and anthropogenic NO_*x*_ emissions inferred with TROPOMI data (TROPOMI-inferred). Modeled season-average profiles are shown during winter (DJF), spring (MAM), summer (JJA), and fall (SON) for days and times matching ozonesonde launches. Shading around sonde and prior emissions profiles represent the maximum and minimum O_3_ at each pressure level. Map shows location of the Trinidad Head launch site.

At the Trinidad Head, California, a location where atmospheric composition is relatively unaffected by local emissions sources and is responsive to trans-Pacific pollution transport (Fig. 11), differences in modeled daily average free-tropospheric O_3_ concentrations can reach +5 or −3ppb. Figure 12 compares CMAQ-modeled vertical O_3_ profiles to observations from 39 ozonesondes launched at Trinidad Head in 2019 (WOUDC, 2019). Relative to the CMAQ simulation using prior emissions, NO_*x*_ emissions updates inferred from OMI and TROPOMI data can improve the model’s ability to reproduce ozonesonde O_3_ distributions measured from the site, in particular during winter and spring, when the discrepancies between modeled and observed concentrations are largest. These impacts on modeled vertical O_3_ profiles are largely driven by changes the modeling framework’s updates to lightning NO_*x*_ emissions. The inferred LNO_*x*_ increases from each satellite improve O_3_ biases, while subsequent anthropogenic updates have smaller impacts, suggesting that biases in O_3_ could be driven by background NO_2_ composition in the model and not solely by long-range transport resulting from anthropogenic emissions.

## 4 Conclusions

In this study, we describe a satellite chemical data assimilation and inverse emissions modeling framework based on the CMAQ hemispheric air quality modeling platform. In the framework, data assimilation adjusts modeled NO_2_ concentrations online using satellite retrievals of tropospheric NO_2_ VCDs. The NO_2_ column changes drive the FDMB inversion, resulting in satellite-constrained top-down emissions estimates. Here, we implement the framework in a NO_*x*_ emissions inversion to separately update 2019 Northern Hemisphere lightning and anthropogenic NO_*x*_ emissions estimates using NO_2_ products from the OMI and TROPOMI satellite instruments. Relative to the modeling platform’s prior emissions derived from regional and global emissions inventories, updates inferred using OMI and TROPOMI observations change average anthropogenic NO_*x*_ emissions by −41 % to +12 % in China, the US, India, Europe, and Mexico. Evaluated against ground-based NO_2_ observations recorded over the US in 2019, the model performs best when using OMI-updated emissions, although a low bias in CMAQ predictions using prior emissions persists into simulations with satellite data assimilation. Compared with US ground-based O_3_ observations, satellite-inferred emissions have mixed impacts on model performance, improving agreement with the measurements during certain months. LNO_*x*_ emissions inferences improve modeled O_3_ when compared against ozonesonde observations across the Northern Hemisphere. The framework’s NO_*x*_ emissions updates also affect model estimates of trans-Pacific O_3_ transport, a source of growing concern in the US, with changes ranging from −3 to +5ppb in simulated O_3_ at a remote West Coast site, resulting from the use of satellite-inferred emissions.

The modeling framework presented has several limitations. The computational cost is greater than that of traditional FDMB inversions due to the assimilation step. However, the computational burden is comparable or less than other satellite assimilation methods such as Kalmanfilter and adjoint 4D variational approaches. In addition, the framework requires minimal code changes to the underlying CTM, so inverse estimates will improve as the underlying air quality model is updated, with little additional effort needed to implement this framework. The global coverage of instruments on polar-orbiting satellites, such as Aura and Sentinel5P, makes the emissions inversions possible but does not allow satellite observations to inform diurnal emissions variations. Upcoming geostationary satellite missions, including GEMS, TEMPO, and Sentinel-4, will provide this capability. Our approach, which balances computational costs and precision in the inversion, is subject to several assumptions. As in all mass-balance-based approaches, our method fully attributes the change in the VCD to emissions changes. To the extent that column differences are due to chemistry or transport and not emissions, this assumption introduces error into mass-balance inversions, including the inversion implemented in our framework. Large changes to the model concentrations resulting from the chemical data assimilation may invalidate assumptions in the subsequent FDMB inversion, leading to biases in the inferred emissions. The FDMB inversion treats each grid cell independently and cannot relate NO_2_ column changes in one grid cell to emissions in another. Although emissions smearing in the approach is mitigated by only analyzing the lower portion of the model column, our emissions changes may be less precise than targeted assimilation methods, such as 4DVAR adjoint-based methods. Further, coarse grid resolution exacerbates biases in modeled NO_2_ columns (Valin et al., 2011) and inferred NO_*x*_ emissions (Sekiya et al., 2021). The air quality model used here does not include stratospheric chemistry, which could affect comparisons against NO_2_ retrievals. Nevertheless, the framework shows the potential to improve air quality model predictions using satellite-derived emissions updates, in particular for regions with uncertain emissions inventories or undergoing rapid emissions changes (Elguindi et al., 2020).

Emissions inversions based on satellite observations can provide valuable information for air quality modeling by addressing the gaps in bottom-up emissions inventories. However, our analysis shows that such inversions and subsequent air quality simulations can be strongly influenced by uncertainties and biases in the satellite data products used. In the analysis conducted, NO_*x*_ emissions inferred from TROPOMI observations appear to be biased low when assessed against those inferred from OMI data and surface and concentration measurements. The bias is consistent with recent research showing a low bias in TROPOMI v1.2 and v1.3 tropospheric columns (Judd et al., 2020; Verhoelst et al., 2021; Li et al., 2021; Van Geffen et al., 2022). The results highlight the importance of efforts to develop robust and consistent satellite data products for use in air quality modeling evaluation, assimilation, and emissions inversions. Ongoing efforts to this end include the MINDS (Lamsal et al., 2020) and the QA4ECV (Boersma et al., 2017) projects. This study also emphasizes the need for longer-term satellite data assimilation and comparisons of established and new satellite data products. The framework introduced here can serve as a generalized tool, with applications beyond those explored in this study, and allows new satellite data products to be incorporated as they become available. As satellite data products evolve and advance, the emissions inferred by the framework will improve.

## Supplementary Material

Supplement1

## Figures and Tables

**Table 1.** Prior emissions and model inputs.

Data	Year	Source
Prior emissions (North America)	2017 EPA platform (v7.1)	Adams (2020)
Prior emissions (China)	2015 Tsinghua University	Zhao et al. (2018)
Prior emissions (rest of hemisphere)	HTAPv2 (2010) projected to 2014 using CEDS scaling factors	Janssens-Maenhout et al. (2015), Hoesly et al. (2018)
Prior emissions (LNO_*x*_ )	2017 GEIA*	Price et al. (1997)
Biomass-burning emissions	2019 FINN*	Wiedinmyer et al. (2011)
Soil NO_*x*_ emissions	2018 CAMS* v2.1 with canopy reduction factor	Granier et al. (2019)
Biogenic emissions	2018 MEGAN*	Guenther et al. (2006)
Meteorology	2019 WRF v4.1.1	Powers et al. (2017)
Satellite observation year	2019 NO_2_ retrievals from OMI and TROPOMI	

* GEIA – Global Emissions Initiative; FINN – Fire Inventory from NCAR; CAMS – Community Atmosphere Modeling System; MEGAN – Model of Emissions of Gases and Aerosols from Nature

**Table 2.** CMAQ model performance evaluated against daily average NO_2_ (DA NO_2_) and maximum 8 h O_3_ concentrations (MDA8 O_3_) observed in 2019 by AQS monitoring sites in the US. Near-road monitors are not considered. Statistics are shown for simulations using prior emissions (Prior), lightning and anthropogenic NO_*x*_ emissions inferred with OMI data (OMI-inferred), and lightning and anthropogenic NO_*x*_ emissions inferred with TROPOMI data (TROPOMI-inferred). Coefficient of determination (*R*), normalized mean error (NME), and normalized mean bias (NMB), relative to AQS observations, are estimated for each CMAQ simulation.

Pollutant	NO_*x*_ emissions	R	NME	NMB

MDA8 O_3_	Prior	0.65	15.9 %	−1.4 %
	OMI-inferred	0.68	15.4 %	3.4 %
	TROPOMI-inferred	0.68	15.3 %	−3.3 %

DA NO_2_	Prior	0.45	62.2 %	−56.9 %
	OMI-inferred	0.52	57.4 %	−49.6 %
	TROPOMI-inferred	0.45	71.7 %	−69.9 %

## Data Availability

NO_*x*_ emissions data derived from this research are available from the authors upon request. Level-2 satellite retrievals are available from NASA’s Goddard Earth Sciences Data and Information Services Center for OMI (https://doi.org/10.5067/Aura/OMI/DATA2017; Krotkov et al., 2019b) and TROPOMI version 1 (https://disc.gsfc.nasa.gov/datasets/S5P_L2__NO2____1/summary; last access: 9 December 2022; https://doi.org/10.5270/S5P-s4ljg54; Copernicus, 2018). TROPOMI retrievals reprocessed to version 2.3.1 are available through the Sentinel-5P data portal (https://data-portal.s5p-pal.com/browser/, last access: 9 December 2022; https://doi.org/10.5270/S5P-9bnp8q8; Copernicus, 2021). WOUDC ozonesonde data, including data at the Trinidad Head, California, launch site, are available through WOUDC at https://doi.org/10.14287/10000008 (WOUDC, 2019). Hourly AQS O_3_ and NO_2_ observations are available from EPA’s Air Data website (https://aqs.epa.gov/aqsweb/airdata/download_files.html, last access: 9 December 2022; U.S. EPA, 2022b). GSI code is available via https://dtcenter.org/community-code/gridpoint-statistical-interpolation-gsi/download (last access: 9 December 2022; DTC, 2018). CMAQ source code is freely available via https://github.com/usepa/cmaq.git (last access: 9 December 2022) and via the U.S. EPA Office of Research and Development (2020; https://doi.org/10.5281/zenodo.4081737).
