# Supplementary material for: Inferring and evaluating satellite-based constraints on NOx emissions estimates in air quality simulations
Source: Atmos Chem Phys. Author manuscript; Available in PMC 2025 Jan 27. (PMC11770562; doi:10.5194/acp-22-15981-2022)
Supplement: Supplement1 [file NIHMS2038940-supplement-Supplement1.pdf]

Supplement of Atmos. Chem. Phys., 22, 15981–16001, 2022
https://doi.org/10.5194/acp-22-15981-2022-supplement
© Author(s) 2022. CC BY 4.0 License.

Supplement of

Inferring and evaluating satellite-based constraints on NO_x emissions estimates in air quality simulations

James D. East et al.

Correspondence to: Fernando Garcia-Menendez (f_garcia@ncsu.edu)

The copyright of individual parts of the supplement might differ from the article licence.

Supplementary information

This file contains 11 figures and 3 tables. Further information is provided on monthly average inferred lightning NO_x (LNOx) emissions (Figs. S1 and S2), analysis increment in the lower and upper troposphere (Fig. S3), inversion framework testing results (Fig. S4), seasonal mean Jacobian (β) values (Fig. S5), impact of NO_x emissions updates on modeled NO_2 VCDs during each season (Figs. S6-S9), emissions increments for TROPOMI v1.2.2 and TROPOMI v2.3.1 (Fig. S10), prior and posterior emissions totals for January 2019 including TROPOMI v2.3.1 posterior (Fig. S11), all CMAQ simulations performed for this study (Table S1), and CMAQ model performance evaluated against ground O_3 observations (Table S2) and NO_2 observations (Table S3).

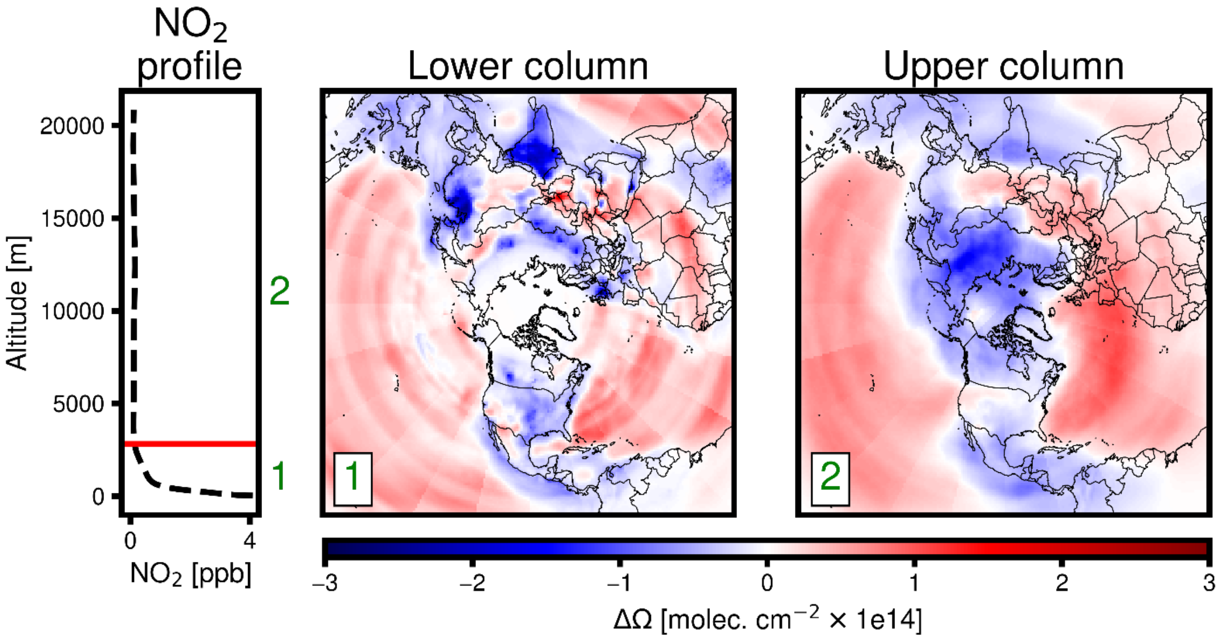

Figure S1: Left panel: Vertical concentration profile of monthly-averaged July NO_2 for the model grid cell containing New York City. Red line indicates the cut-off used for lower troposphere in the emissions inversion. Center and right panels: Monthly average difference in modeled NO_2 columns between simulations with and without assimilated NO_2 satellite data (**$\Delta\Omega$**), for lower (1) and upper (2) column regions defined by the emissions inversion cutoff. **$\Delta\Omega$** is shown for one iteration of assimilating July 2019 TROPOMI NO_2 with background errors for the boundary layer and LNO_x emissions updates applied.

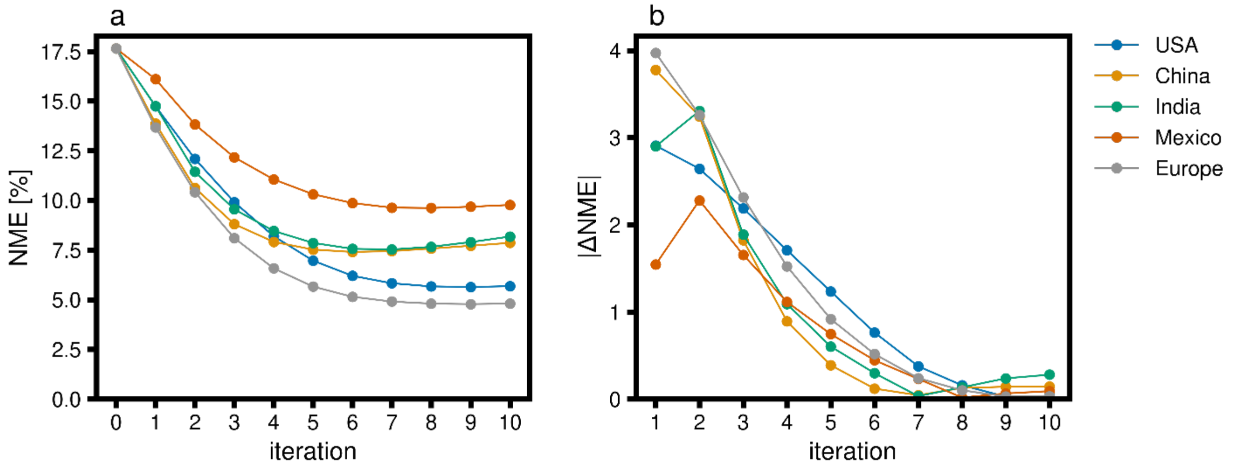

Figure S2: Synthetic inversion test results showing (a) NME (%) by region for each iteration of FDMB, and (b) change in NME (%) by region for each iteration of FDMB. Synthetic observations are created by a simulation initialized with a uniform 15% reduction in anthropogenic NO_x emissions. Errors are computed using the VCDs within five regions of the Northern Hemisphere including only grid cells that are included in the inversion according to the filtering criteria described in Sect. 2.5.

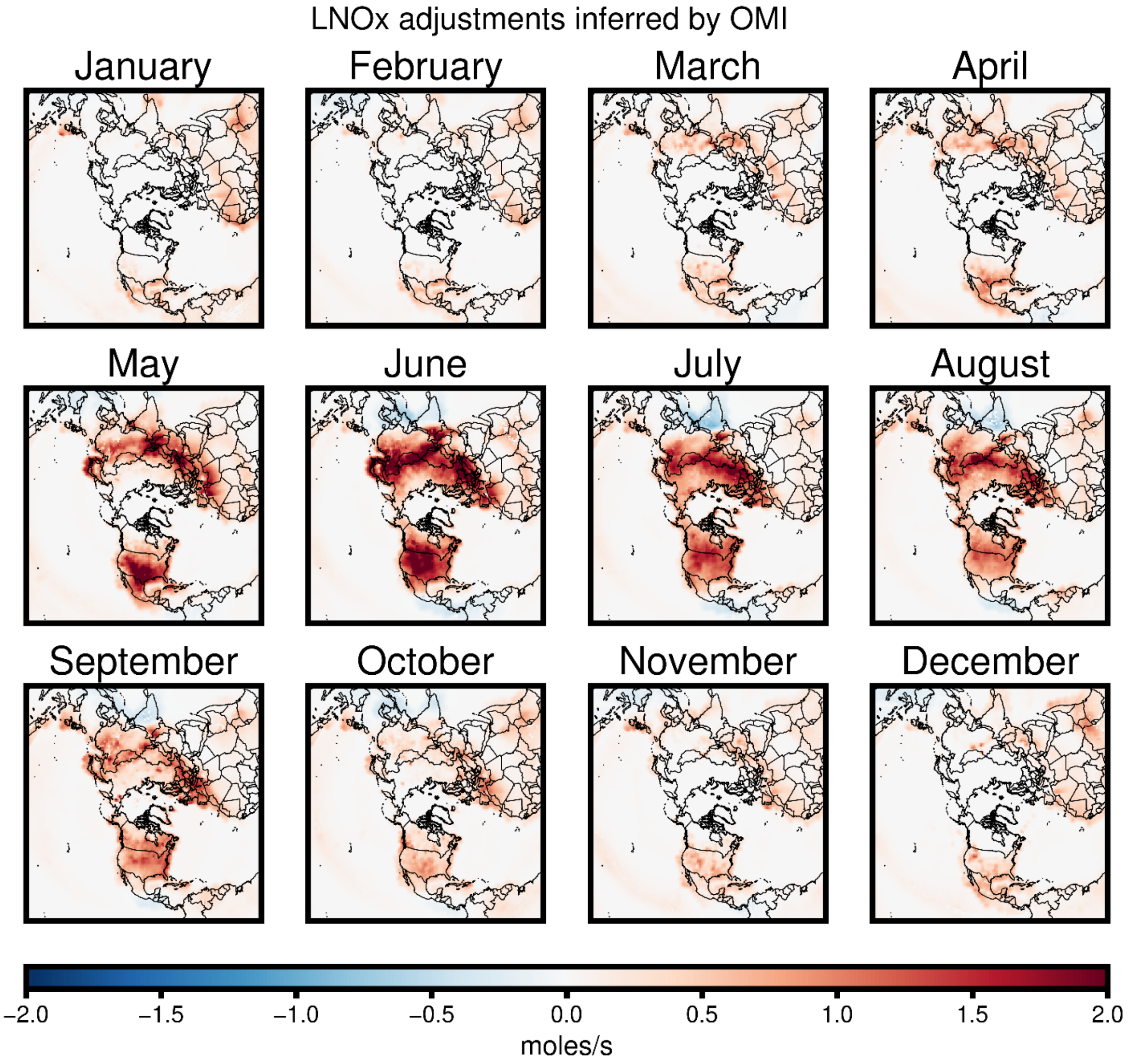

Figure S3: Monthly-average 2019 LNOx emissions changes inferred from OMI observations.

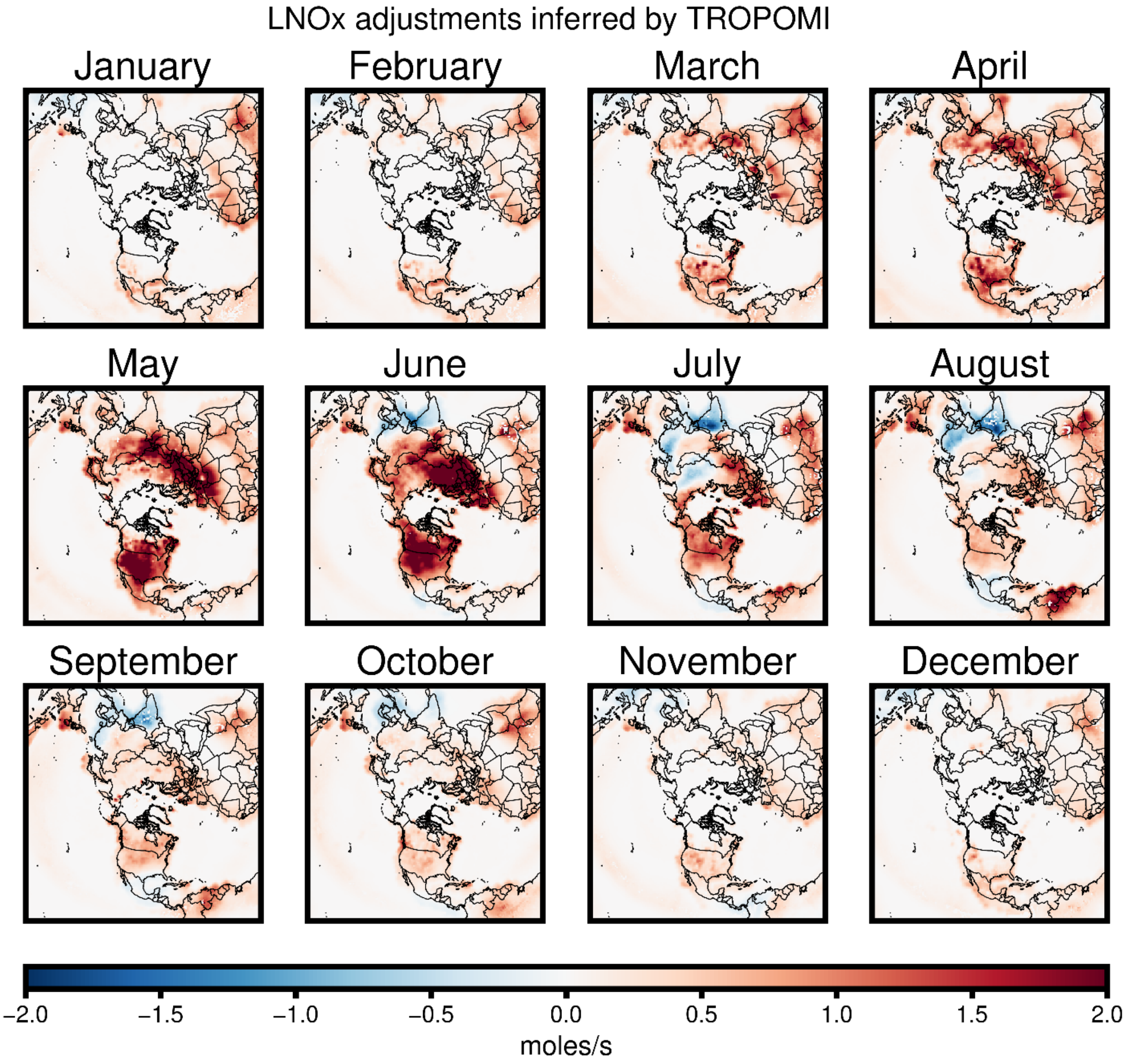

Figure S4: Monthly-average 2019 LNOx emissions changes inferred from TROPOMI observations.

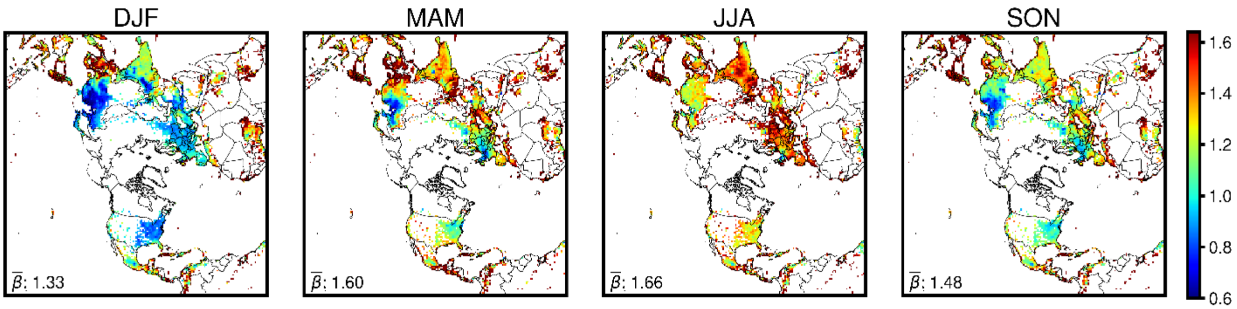

Figure S5: Seasonal mean Jacobian ($\boldsymbol{\beta}$) relating changes in NO_x emissions to changes in the lower tropospheric NO_2 vertical column. $\boldsymbol{\beta}$ shown for winter (DJF), spring (MAM), summer (JJA), and fall (SON). Domain-average values across ($\bar{\boldsymbol{\beta}}$) are indicated on each map.

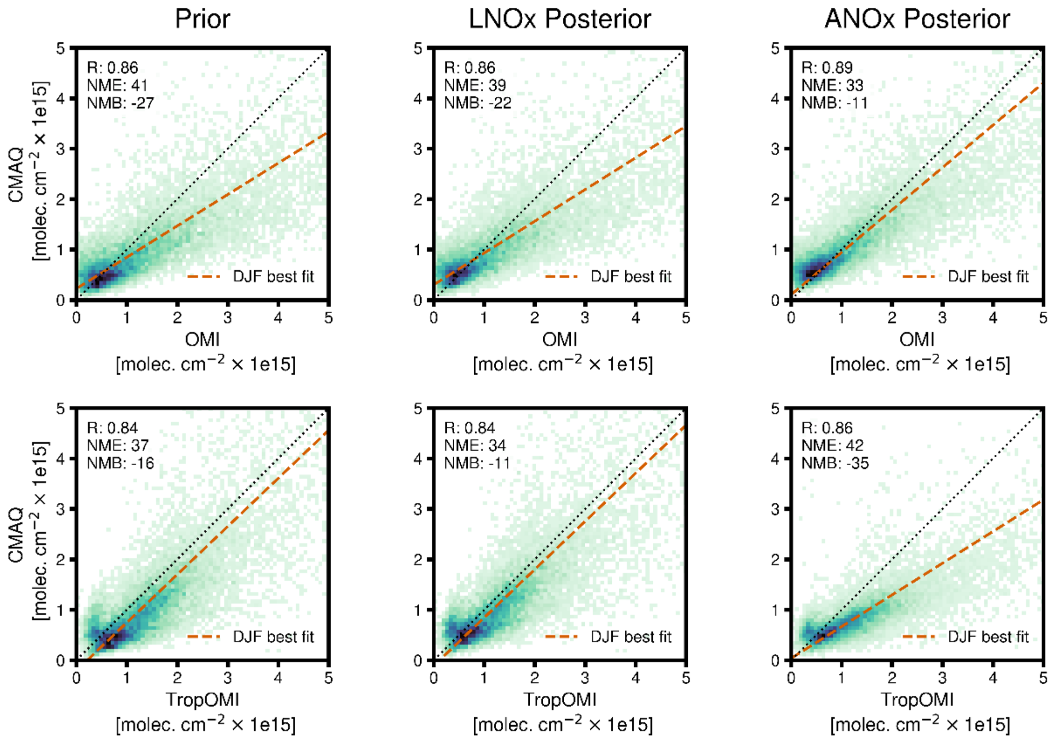

Figure S6: Impact of NO_X emissions updates on modeled NO_2 VCD during winter (December, January, and February). Plots compare 2019 monthly-average CMAQ-modeled NO_2 VCD at each domain grid cell in which NO_X emissions were updated by the inverse modeling system against OMI and TROPOMI tropospheric NO_2 VCD retrievals averaged in each cell. Modeled NO_2 VCD using prior emissions (Prior), inferred LNOx emissions (LNOx posterior), and inferred lightning and anthropogenic NOx emissions (ANOx posterior) are each compared with NO_2 VCD retrievals. Top row plots compare retrievals and modeled VCD based on OMI observations, while bottom row plots compare retrievals and modeled VCD based on TROPOMI observations. Linear regression line, coefficient of determination (R), normalized mean error (NME), and normalized mean bias (NMB), relative to tropospheric NO_2 VCD retrievals, are shown for each CMAQ simulation.

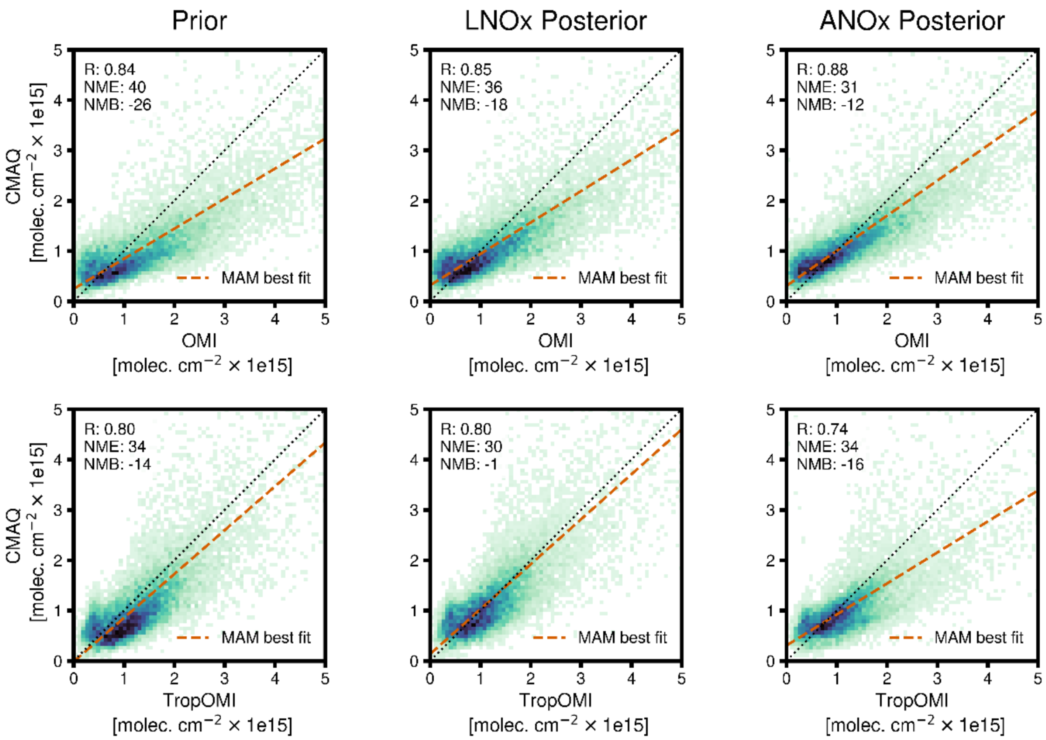

Figure S7: Impact of NO_X emissions updates on modeled NO_2 VCD during spring (March, April, and May). Plots compare 2019 monthly-average CMAQ-modeled NO_2 VCD at each domain grid cell in which NO_X emissions were updated by the inverse modeling system against OMI and TROPOMI tropospheric NO_2 VCD retrievals averaged in each cell. Modeled NO_2 VCD using prior emissions (Prior), inferred LNOx emissions (LNOx posterior), and inferred lightning and anthropogenic NOx emissions (ANOx posterior) are each compared with NO_2 VCD retrievals. Top row plots compare retrievals and modeled VCD based on OMI observations, while bottom row plots compare retrievals and modeled VCD based on TROPOMI observations. Linear regression line, coefficient of determination (R), normalized mean error (NME), and normalized mean bias (NMB), relative to tropospheric NO_2 VCD retrievals, are shown for each CMAQ simulation.

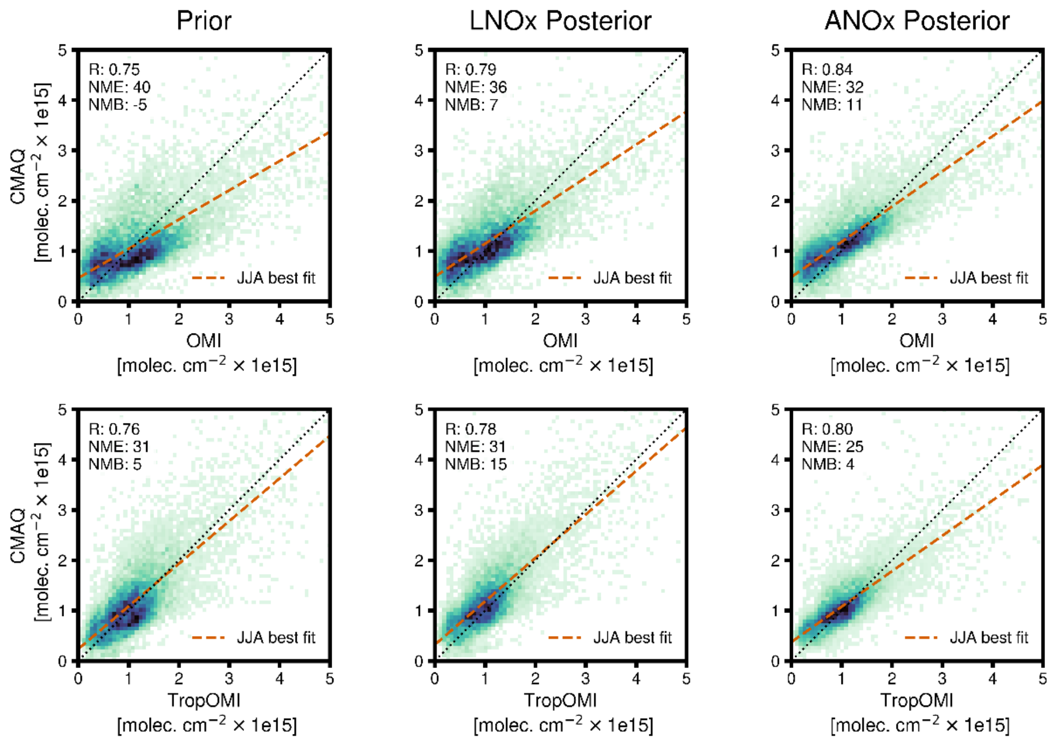

Figure S8: Impact of NO_X emissions updates on modeled NO_2 VCD during summer (June, July, and August). Plots compare 2019 monthly-average CMAQ-modeled NO_2 VCD at each domain grid cell in which NO_X emissions were updated by the inverse modeling system against OMI and TROPOMI tropospheric NO_2 VCD retrievals averaged in each cell. Modeled NO_2 VCD using prior emissions (Prior), inferred LNOx emissions (LNOx posterior), and inferred lightning and anthropogenic NOx emissions (ANOx posterior) are each compared with NO_2 VCD retrievals. Top row plots compare retrievals and modeled VCD based on OMI observations, while bottom row plots compare retrievals and modeled VCD based on TROPOMI observations. Linear regression line, coefficient of determination (R), normalized mean error (NME), and normalized mean bias (NMB), relative to tropospheric NO_2 VCD retrievals, are shown for each CMAQ simulation.

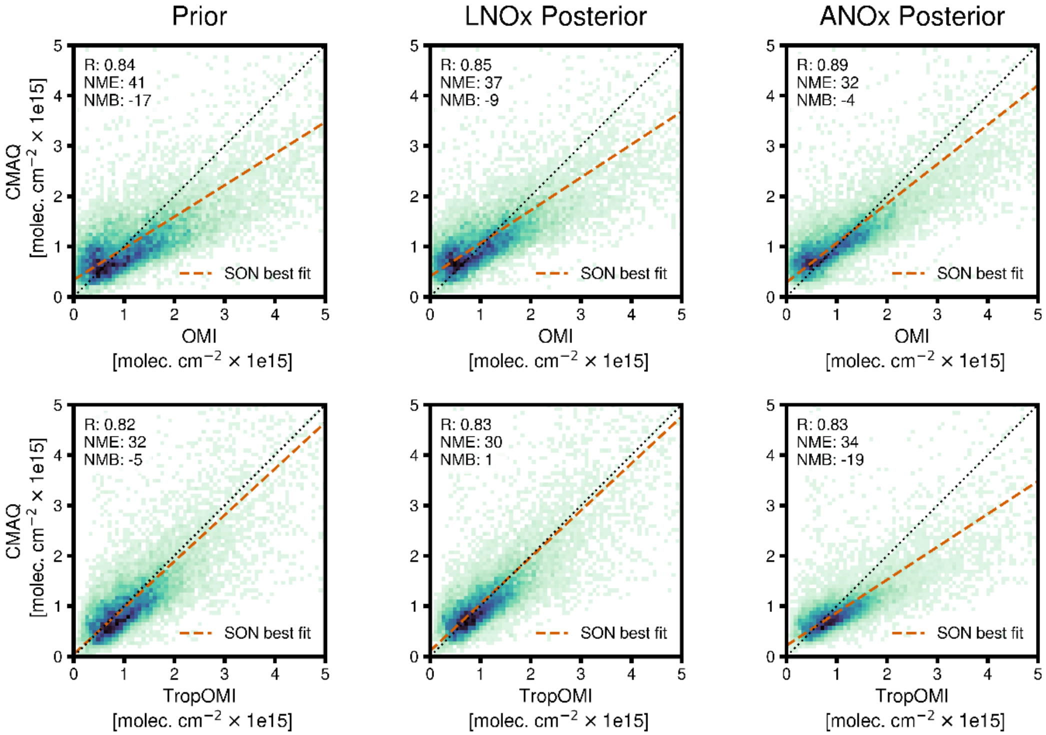

Figure S9: Impact of NO_X emissions updates on modeled NO_2 VCD during autumn (September, October, and November). Plots compare 2019 monthly-average CMAQ-modeled NO_2 VCD at each domain grid cell in which NO_X emissions were updated by the inverse modeling system against OMI and TROPOMI tropospheric NO_2 VCD retrievals averaged in each cell. Modeled NO_2 VCD using prior emissions (Prior), inferred LNOx emissions (LNOx posterior), and inferred lightning and anthropogenic NOx emissions (ANOx posterior) are each compared with NO_2 VCD retrievals. Top row plots compare retrievals and modeled VCD based on OMI observations, while bottom row plots compare retrievals and modeled VCD based on TROPOMI observations. Linear regression line, coefficient of determination (R), normalized mean error (NME), and normalized mean bias (NMB), relative to tropospheric NO_2 VCD retrievals, are shown for each CMAQ simulation.

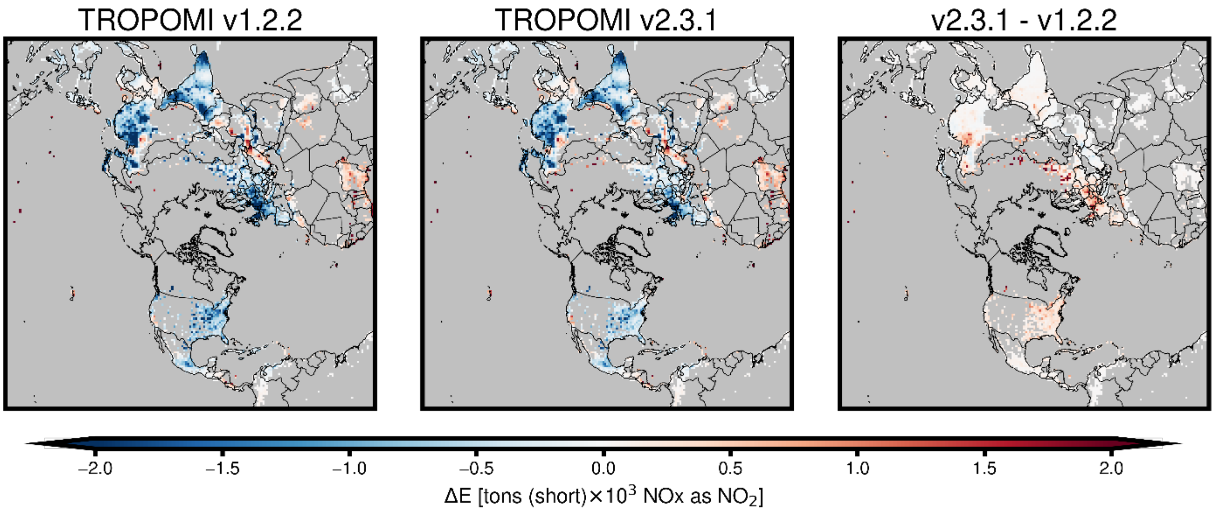

Figure S10: Anthropogenic NO_X emissions increment for January 2019 inferred from TROPOMI version 1.2.2 and version 2.3.1 NO_2 observations, and difference in the analysis increment based on the two datasets.

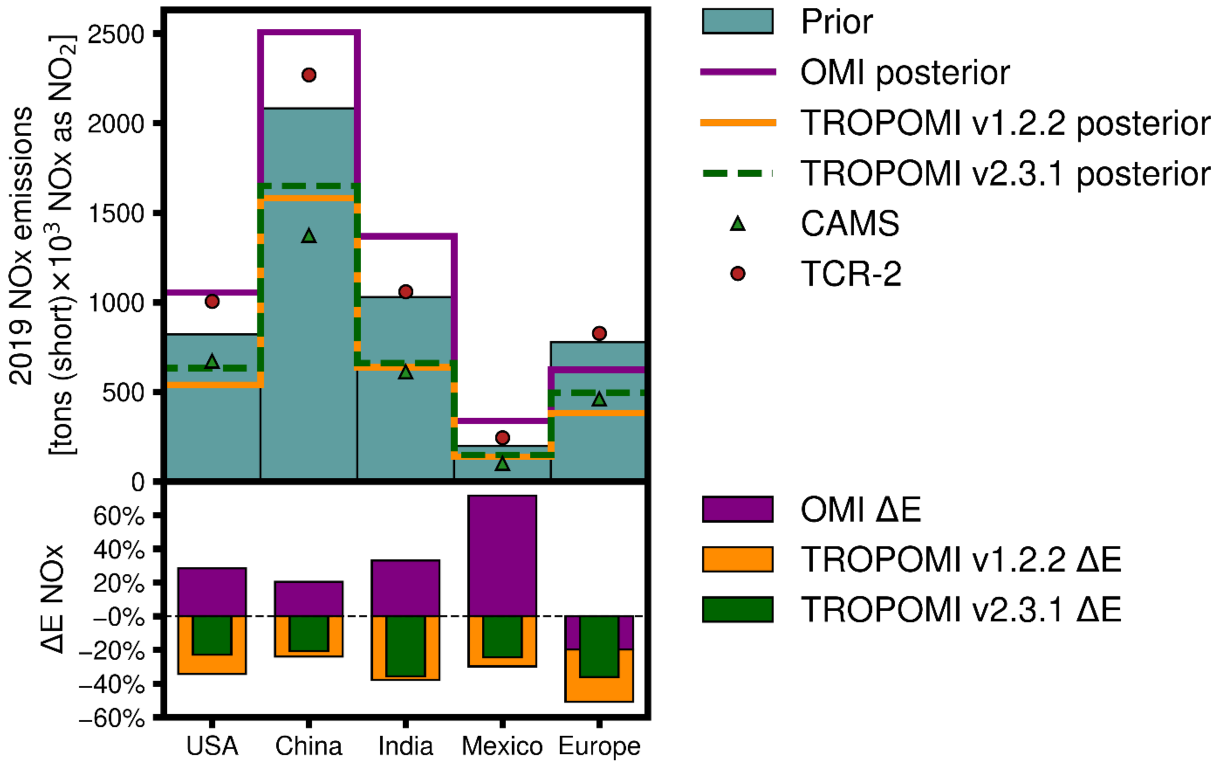

Figure S11: Prior and satellite-inferred January 2019 anthropogenic NOx emissions in select global regions. Top plot shows total emissions (as NO_2) from prior emissions estimates, inference with TROPOMI version 1.2.2 and 2.3.1 observations (TROPOMI v1.2.2 and v2.3.1 posterior), inference with OMI (OMI posterior), and CAMS or TCR-2 inventories in the U.S., China, India, Mexico, and Europe. The bottom plot shows the percent change ($\Delta E\ NO_x$) inferred with OMI or TROPOMI data, relative to prior emission estimates, for each region.

Table S1: Hemispheric CMAQ simulations conducted as part of this study

**Simulation**	**Satellite data assimilated**	**Anthropogenic Emissions (initial)**	**LNO_x emissions**	**Emissions perturbation**	**Iteration**	**Used for emissions adjustment**
Base	-	Prior	Prior	-	No	-
No LNO_x	-	Prior	Prior	0% LNOx	No	-
OMI LNO_x update	OMI NO_2	Prior	Prior	-	No	LNO_x
TROPOMI LNO_x update	TROPOMI NO_2	Prior	Prior	-	No	LNO_x
OMI Base LNO_x	-	Prior	Posterior (OMI)	-	No	-
OMI ANO_x reduced	-	Prior	Posterior (OMI)	ANOx 15% reduced	No	-
OMI ANO_x update	OMI NO_2	Prior	Posterior (OMI)	-	Yes	ANO_x
OMI posterior	-	Posterior (OMI)	Posterior (OMI)	-	No	-
TROPOMI Base LNO_x	-	Prior	Posterior (TropOMI)	-	No	-
TROPOMI ANOx reduced	-	Prior	Posterior (TropOMI)	ANOx 15% reduced	No	-
TROPOMI ANOx update	TROPOMI NO_2	Prior	Posterior (TropOMI)	-	Yes	ANO_x
TROPOMI posterior	-	Posterior (TropOMI)	Posterior (TropOMI)	-	No	-

Table S2: CMAQ model performance evaluated against daily maximum 8-hour O_3 concentrations (MDA8 O_3) observed in 2019 by 1,218 AQS monitoring sites in the U.S., during winter (Dec.-Feb.), spring (Mar.-May), summer (Jun.-Aug.), and fall (Sep.-Nov.) months. Near-road monitors are not considered. Statistics are shown for simulations using prior emissions (Prior), lightning and anthropogenic NOx emissions inferred with OMI data (OMI-inferred), and lightning and anthropogenic NOx emissions inferred with TROPOMI data (TROPOMI-inferred). Coefficient of determination (R), normalized mean error (NME), and normalized mean bias (NMB), relative to AQS observations, are estimated for each CMAQ simulation.

Season	NO_x emissions	R	NME	NMB
Winter	Prior	0.60	15.8%	-4.8%
	OMI-inferred	0.61	15.0%	-0.5%
	TROPOMI-inferred	0.56	16.0%	-3.0%
Spring	Prior	0.53	15.9%	-10.2%
	OMI-inferred	0.57	13.4%	-4.9%
	TROPOMI-inferred	0.55	16.6%	-11.6%
Summer	Prior	0.58	17.2%	6.8%
	OMI-inferred	0.65	17.8%	11.8%
	TROPOMI-inferred	0.67	14.4%	3.2%
Fall	Prior	0.73	14.5%	2.3%
	OMI-inferred	0.74	15.1%	6.6%
	TROPOMI-inferred	0.73	14.3%	-1.0%

Table S3: CMAQ model performance evaluated against daily 24-h average NO_2 concentrations observed in 2019 by 1,218 AQS monitoring sites in the U.S., during winter (Dec.-Feb.), spring (Mar.-May), summer (Jun.-Aug.), and fall (Sep.-Nov.) months. Near-road monitors are not considered. Statistics are shown for simulations using prior emissions (Prior), lightning and anthropogenic NOx emissions inferred with OMI data (OMI-inferred), and lightning and anthropogenic NOx emissions inferred with TROPOMI data (TROPOMI-inferred). Coefficient of determination (R), normalized mean error (NME), and normalized mean bias (NMB), relative to AQS observations, are estimated for each CMAQ simulation.

Season	NO_x emissions	R	NME	NMB
Winter	Prior	0.42	64.8%	-60.5%
	OMI-inferred	0.47	57.4%	-49.3%
	TROPOMI-inferred	0.47	76.3%	-75.4%
Spring	Prior	0.45	64.2%	-59.7%
	OMI-inferred	0.48	57.5%	-48.7%
	TROPOMI-inferred	0.48	70.9%	-69.0%
Summer	Prior	0.37	57.2%	-49.0%
	OMI-inferred	0.39	55.4%	-46.0%
	TROPOMI-inferred	0.43	63.3%	-59.5%
Fall	Prior	0.41	60.8%	-55.5%
	OMI-inferred	0.53	58.5%	-53.1%
	TROPOMI-inferred	0.44	72.4%	-71.0%
